# Hartnup disease-causing SLC6A19 mutations lead to B0AT1 aberrant trafficking and ACE2 mis-localisation implicating the endoplasmic reticulum protein quality control

**DOI:** 10.3389/fcell.2025.1589534

**Published:** 2025-08-07

**Authors:** Nesreen F. Alkhofash, Bassam R. Ali

**Affiliations:** ^1^ Department of Genetics and Genomics, College of Medicine and Health Sciences, United Arab Emirates University, Al-Ain, United Arab Emirates; ^2^ ASPIRE Precision Medicine Research Institute Abu Dhabi, United Arab Emirates University, Al-Ain, United Arab Emirates

**Keywords:** ACE2, B0AT1, ACE2 variants, B0AT1 variants, sub-cellular localization, molecular modulators, endoplasmic reticulum, ER-retained mutant variants

## Abstract

**Introduction:**

The interaction between angiotensin-converting enzyme 2 (ACE2) and the sodium-dependent Broad neutral Amino acid Transporter 1 (B0AT1), encoded by the *SLC6A19* gene, is increasingly recognized as pivotal in both physiological and pathological contexts. B0AT1 facilitates neutral amino acid transport and nutrient absorption, while ACE2 regulates vascular homeostasis and inflammation through the renin-angiotensin system. Mutations in *SLC6A19* are implicated in Hartnup disease, a metabolic disorder characterized by defective amino acid transport. However, the cellular mechanisms underlying Hartnup disease-causing mutations' impact on B0AT1 and ACE2 function remain unclear.

**Methods:**

This study evaluated the subcellular localization and trafficking of 18 Hartnup disease-causing B0AT1 variants using experimental approaches including biochemical assays and In Silico analysis. The impact of these variants on ACE2 trafficking and plasma membrane targeting was also assessed to elucidate their interplay.

**Results:**

Nine B0AT1 variants (R57C, G93R, R95P, R178Q, L242P, G284R, S303L, D517G, P579L) were found to be retained in the endoplasmic reticulum, impairing their trafficking to the plasma membrane. These variants were distributed across multiple B0AT1 structural domains. Importantly, several of these ER-retained variants, particularly R178Q and S303L, significantly disrupted ACE2 intracellular trafficking and its localization to the plasma membrane, indicating a direct effect on ACE2 subcellular targeting.

**Discussion:**

The findings reveal that Hartnup disease-causing mutations can lead to ER retention of B0AT1, which in turn has a variable effect on ACE2 trafficking. This disruption likely contributes to Hartnup disease pathogenesis by impairing amino acid transport and may influence ACE2-mediated physiological functions beyond the renin-angiotensin system. Understanding these molecular mechanisms enhances insight into ACE2-B0AT1 interactions and could inform future therapeutic strategies and biomarker development for related disorders. Further research is needed to explore these pathways and their implications in disease.

## 1 Introduction

The *SLC6A19* gene encodes the Broad neutral Amino acid Transporter 1 (B0AT1), which functions as the primary neutral amino acid transporter from the luminal compartment to the intracellular space of the intestine and renal proximal tubules in a sodium (Na+)-dependent manner (Doyle and McGivan, 1992; Soriano-García et al., 1998; Stevens et al., 2021). B0AT1 is predominantly expressed in the kidney and intestine, with lower expression levels in other tissues such as the pancreas, stomach, liver, duodenum, and ileocecum (Kleta et al., 2004; Seow et al., 2004; Tian et al., 2018). Mutations in the *SLC6A19* gene have been identified as the cause of autosomal-recessive Hartnup disorder (OMIM 234500), a rare genetic disorder characterized by impaired absorption of neutral amino acids in the small intestine and kidneys (Kleta et al., 2004; Seow et al., 2004). Although clinical symptoms are only observed in rare cases of the disorder some are characterised as skin rashes, neurological symptoms, and potentially psychiatric disorders, such as seizures, attention-deficit hyperactivity disorder, intellectual disability, pellagra and ataxia (Bröer, 2009; Camargo et al., 2008; Camargo et al., 2020; Cheon et al., 2010; Haijes et al., 2020; Kleta et al., 2004; Seow et al., 2004; Wang et al., 2022; Wilcken et al., 1977). The disorder is thought to develop due to hindered neutral amino acid transport across cells. Biochemically, it is characterized by decreased reabsorption and increased excretion of neutral amino acids such as alanine, serine, threonine, valine, leucine, isoleucine, phenylalanine, tyrosine, asparagine, glutamine, tryptophan, histidine and citrulline (Haijes et al., 2020; Wang et al., 2022).

On the other hand, Molecular docking models and gut-lung axis studies of COVID-19 show that Angiotensin-Converting Enzyme-2 (ACE2) binds and dimerizes with B0AT1 (Stevens et al., 2021; Yan et al., 2020). The ACE2 protein has recently been shown to be used as the receptor for cell membrane attachment and entry of the SARS-CoV-2 virus (Walls et al., 2020; Wrapp et al., 2020; Zhang et al., 2022). This ACE2-B0AT1 complex is thought to influence SARS-CoV-2’s preference for the gastrointestinal (GI) tract (Kuba et al., 2005; Li et al., 2003). ACE2 is usually localised at the plasma membrane (Alkhofash and Ali, 2024; Stevens et al., 2021). A systematic review including more than 40 clinical studies with over 18,000 patients found that GI problems are common in COVID-19 patients (Silva and França, 2020). Additionally, in *ace2* knockout mice, B0AT1 was absent from the intestinal brush-border membrane, although it was present in the kidney, demonstrating a tissue-specific requirement of this auxiliary protein for B0AT1 trafficking to the plasma membrane (Camargo et al., 2009). In enterocytes, ACE2 is co-expressed with B0AT1. Together, B0AT1 and ACE2 form a quaternary complex, a dimer of heterodimers (Kowalczuk et al., 2008; Stevens et al., 2021; Xiao et al., 2021), referred to as the [ACE2:B0AT1]_2_ super-complex due to its significantly large size and heavy molecular weight (Yan et al., 2020). The [ACE2-B0AT1]_2_ super-complex 3D structure is available in Protein Data Bank with IDs 6M17 and 6M18. It has been shown that [ACE2-B0AT1] heterodimer pairs act as functional units within a 4-part structure (Barnes et al., 2020; Stevens et al., 2021). Stevens et al. experimentally confirmed the [ACE2-B0AT1]_2_ super-complex using purified lyophilized enterocyte brush-border membrane vesicles. Their findings included Na + -dependent neutral amino acid transport activity on the apical side of intact membranes. This activity was carried out by a protein complex with a molecular weight of 183.7 ± 16.8 kDa, representing one heterodimer of [ACE2:B0AT1]. Each thermodynamically stable [ACE2:B0AT1] heterodimer functional unit was responsible for the transport activity within the full quaternary structural complex: a ∼345 kDa [ACE2:B0AT1]_2_ dimer of heterodimers (Stevens et al., 2021).

**FIGURE 1:**
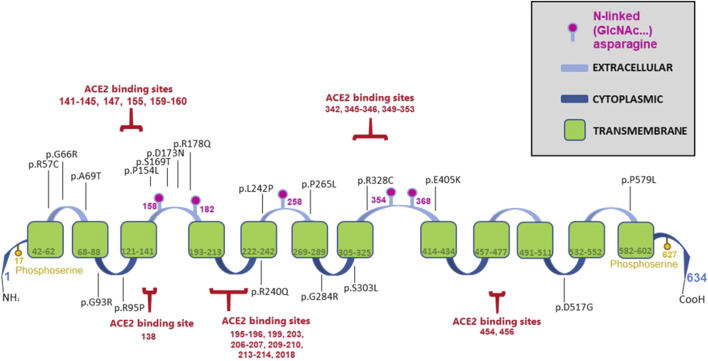
Schematic diagram of the B0AT1 protein illustrating its various domains, post-translational modification sites, ACE2 binding sites and the selected 18 Hartnup disease-causing missense mutations studied in this manuscript.

Hartnup disease-causing mutations in B0AT1 are distributed across the protein’s multidomain structure, as illustrated in Figure 1. The protein is highly hydrophobic with twelve transmembrane α-helices arranged in a LeuT-fold, with both its N- and C-terminals located on the intracellular cytoplasmic side of the plasma membrane (Yan et al., 2020; Zhang et al., 2022). B0AT1 interacts directly with ACE2, a protein composed of an extracellular peptidase domain (PD) and a collectrin-like domain (CLD), which terminates in a single transmembrane α-helix. ACE2 interacts with an extended loop linking transmembrane helices 7 and 8 in B0AT1, enabling cell surface expression (Bröer and Gether, 2012; Danilczyk et al., 2006; Kowalczuk et al., 2008; Yan et al., 2020).

A key indicator of dysfunctional B0AT1 is elevated levels of neutral amino acids in the patient’s urine compared to normal individuals (Belanger et al., 2018; Jiang et al., 2015). This is often accompanied by bi-allelic mutations in the *SLC6A19* gene (Kleta et al., 2004; Garrido et al., 2022; Seow et al., 2004; Wang et al., 2022). The 18 disease-causing mutations (Figure 1) analysed in this study were retrieved from the literature and the GenomAD database (Azmanov et al., 2007; Azmanov et al., 2008; Bröer, 2009; Camargo et al., 2009; Cheon et al., 2010; Chiba et al., 2014; Gupta et al., 2020; Kanchi et al., 2014; Kleta et al., 2004; Seow et al., 2004; Wang et al., 2022; Zheng et al., 2009). Site-directed mutagenesis was employed to generate *SLC6A19* cDNAs carrying each of the indicated mutations in a mammalian expression vector. This study investigated if these mutations disrupted the subcellular localization and translocation of B0AT1 to the plasma membrane as a potential mechanism to their dysfunction and disease pathogenesis.

The subcellular localizations of Hartnup-casing B0AT1 variants are not well characterized in literature. Furthermore, there are no biochemical or localization studies describing the outcome of their interactions with wild type ACE2. It is important to note that both proteins interact directly, creating a heterodimer, and two heterodimers dimerize to form a super-complex that interacts with S1 of Coronaviruses (Camargo et al., 2009; Camargo et al., 2020; Ferreira-Duarte et al., 2020; Scalise and Indiveri, 2020; Stevens et al., 2021; Yan et al., 2020; Zhang et al., 2022). Our study aimed to provide a better understanding of the pathogenesis of representative Hartnup-causing missense variants of B0AT1 and their potential effects on B0AT1 interaction with ACE2, as well as their impact on subcellular trafficking. Our findings indicate that 9 out of the 18 analysed B0AT1 mutations associated with Hartnup disease (R57C, G93R, R95P, R178Q, L242P, G284R, S303L, D517G, and P579L) lead to substantial retention of the mutated B0AT1 transporter within the endoplasmic reticulum (ER), suggesting impaired trafficking to the plasma membrane. Additionally, our data show that two (R178Q, S303L) of the nine ER-retained mutants have a significant impact on ACE2 intracellular trafficking and subcellular targeting to the plasma membrane. These findings provide further insights into the pathogenesis of Hartnup disease, which may potentially contribute to the development of novel treatments.

## 2 Materials and methods

### 2.1 Cell lines and cell culture

HeLa and HEK293T cells were used in this study and were cultured in Dulbecco’s Modified Eagle Medium (Gibco) as previously described (Alkhofash and Ali, 2024). Culture media were supplemented with 10% fetal bovine serum (Gibco), antibiotic–antimycotic (Gibco) and cells were incubated at 37 C with 5% CO2.

### 2.2 Mutagenesis primers design and the generation of B0AT1 missense variants by site-directed mutagenesis

The FLAG-tagged plasmids of Human B0AT1 wild type (NM_001003841) (RC212948), and Human ACE2 wild type (NM_021804) (RC208442) were purchased from OriGene Inc. The selected 18 B0AT1 missense variants, reported to be disease-causing (Supplementary Table S1), were generated by Quick-Change site-directed mutagenesis with Pfu Ultra High-Fidelity DNA polymerase (Stratagene Inc) with the wild type B0AT1 construct (RC212948) being used as a template. ACE2 has been mutated (c.C2542T) to remove the Myc-DDK tags from the original construct by creating a stop codon before the FLAG tag sequence. This modification allowed us to distinguish between the two proteins, by using anti-ACE2 specific antibodies that are of high quality and sensitivity. The primers for mutagenesis were designed in PrimerX software (https://www.bioinformatics.org/primerx/) and custom-made by Metabion International AG (https://www.metabion.com/). The desired variants were confirmed by Sanger DNA sequencing using the ABI 3130xl automated fluorescent Genetic Analyzer (Applied Biosystems). Clustal Omega software (https://www.ebi.ac.uk/Tools/msa/clustalo/) was used for sequence alignments.

### 2.3 Immunofluorescence and confocal microscopy

HeLa cells were seeded on sterilized cover slips in 24-well plates. When the cells reached approximately 60% confluency, they were co-transfected using FuGENE® HD Transfection Reagent (Promega, Cat# E2311) in 3:1 ration with the selected plasmid(s) as described in the figure legends with a cDNA total of 0.5 μg (FLAG tagged B0AT1, untagged-ACE2 and GFP-tagged HRas plasmid in the following ration 4:1:0.5). GFP-HRas is a well-established plasma membrane marker (Ali et al., 2010; Badawi et al., 2022). Twenty-four hours after transfection, the cells were washed three times with phosphate-buffered saline (PBS) and then fixed in absolute methanol at room temperature for 5 min. After washing three times with PBS, nonspecific antigen binding was blocked with 1% bovine serum albumin (Sigma Aldrich, St. Louis, MO) for 1 h at room temperature. Co-staining with anti-FLAG primary antibody (1:100 Cell Signaling) and anti-Calnexin (1:50 Santa Cruz Biotechnology) was performed for 1 h in the dark at room temperature. The slides then washed three times with PBS and incubated with the respective secondary antibody (Thermo Fischer Scientific) for 45 min at room temperature in the dark. Finally, cells were washed with PBS and mounted with fluorescence mounting medium (Dako). Images were acquired using a ×100 objective Nikon confocal Eclipse 80I microscope (Nikon Instruments Inc.). The methodology used has been described in details previously (Alkhofash and Ali, 2024; Badawi et al., 2022).

### 2.4 Colocalization analysis of B0AT1 using CellProfiler

To evaluate the subcellular localization of B0AT1 (red signal), a comprehensive colocalization analysis was performed using CellProfiler (version 4.2.8), an open-source image analysis software. Fluorescence microscopy images of cells expressing wild type and variant forms of B0AT1 were analyzed in relation to an endoplasmic reticulum (ER) marker (blue signal) and a plasma membrane marker (green signal). Images are in Supplementary Figure S1 while the generated table of computational assay outcome is in the Supplementary Table S2.

The “MeasureColocalization” module in CellProfiler was used to quantify spatial relationships between red and each reference channel (green and blue) using the following metrics:

• Pearsons’ Correlation Coefficient assesses the linear relationship between pixel intensities in the two channels, indicating how similarly their signals vary across the image.
• Manders’ Coefficients (M1 and M2) calculate the fraction of one signal that overlaps with the other, regardless of intensity variation, indicating the extent of spatial co-occurrence.
• Manders’ Coefficient (Costes method) applies automated thresholding to reduce background and identify statistically significant colocalization.
• Robust Weighted Colocalization (RWC) Coefficient provides a weighted measure of overlap, integrating both signal intensity and spatial proximity.
• Overlap Coefficient quantifies the degree of shared spatial signal between channels.
• Slope from regression analysis reflects the proportionality of intensity values between the channels.

For each cell or field, these metrics were computed between red-green (B0AT1–plasma membrane) and red-blue (B0AT1–ER) pairs. To determine the likely localization of B0AT1 (wild-type and variants), the following decision criteria were applied:

• If red-blue correlation (Pearsons’ and/or RWC Coefficient) was greater than red-green correlation, the signal was interpreted as ER-retained.
• If red-green correlation exceeded red-blue, the signal was classified as plasma membranal.

This standardized pipeline was applied uniformly across all tested constructs, including the wild-type B0AT1 and its mutant variants, to assess the impact of mutations on intracellular trafficking and membrane targeting.

### 2.5 SDS-PAGE immunoblotting

HEK293T cells cultured in 6 well-plates were transfected using FuGENE® HD Transfection Reagent (Promega, Cat# E2311) in 3:1 ration with the selected plasmid(s) as described in the figure legends with a cDNA total of 1.0 μg (FLAG tagged B0AT1 and untagged-ACE2 plasmid in several ratios as indicted in Western blot results). Approximately 18 h post cells were harvested and lysed in the FIVEphoton Biochemicals Transmembrane Protein Extraction Reagent (Fivephoton Biochemicals, model# tmPER-50TM), supplemented with a protease inhibitor cocktail (Sigma fast protease inhibitor cocktail, Cat#P8340). The collected protein lysates were quantified using a colorimetric bicinchoninic acid (BCA) protein assay (Pierce, Cat# 23225). Thirty µg of total protein lysates were resolved on 10% SDS-PAGE gels, followed by semi-dry transfer to PVDF membrane. The transfer buffer, containing 15% methanol and supplemented with 0.06% SDS, was used, and the transfer was performed for 40 min. The membrane blots were then blocked to prevent nonspecific binding with 1% bovine serum albumin (Sigma Aldrich, St. Louis, MO) for 1 h at room temperature. The membranes were incubated overnight with the primary antibodies: Anti-FLAG (1:800, Cell Signalling), anti- ACE2 (1:400, Invitrogen), and anti-β-actin (1:1000, Santa Cruz Biotechnology), followed by incubation with the corresponding secondary antibodies (Sigma Aldrich, St. Louis, MO) for 1 h at room temperature. This was after washing the blots three times with 1xTBST (Tris-buffered saline with 0.1% Tween®). The membranes were then incubated with Enhanced Chemiluminescence Plus Reagent (Pierce) and developed using the Typhoon FLA 9500 imager (GE Healthcare Bio sciences, Piscataway, NJ, United States). Alternatively, the membranes were incubated with SuperSignal™ West Pico PLUS Chemiluminescent Substrate (Thermo Fischer Scientific, cat #34579) and developed through Azure Imaging Systems (Azure Biosystems Inc., Dublin, CA, United States). Western blots were analysed and contrast-enhanced using ImageJ software.

### 2.6 N-glycosylation endoglycosidase H (endo H) sensitivity and resistance assay

HEK293T cells were seeded in 6-well plates and transfected with wild-type or mutant B0AT1 plasmids. Cells were harvested, lysed in the FIVEphoton Biochemicals Transmembrane Protein Extraction Reagent (Fivephoton Biochemicals, model# tmPER-50TM), supplemented with a protease inhibitor cocktail (Sigma fast protease inhibitor cocktail, Cat# P8340). Protein lysates were collected and quantified as previously described. The Sigma kit was used for the endoglycosidase H (Endo H) sensitivity and resistance assay, following the manufacturer’s protocol (Sigma, Cat# A0810) as previously described (Badawi et al., 2022; Badawi et al., 2023). Briefly, cell lysates were denatured in a denaturation buffer (Sigma Aldrich, St. Louis, MO, Cat# S4927) at 60°C for 5 min, and all the subsequent steps were carried out according to the manufacturer’s protocol. Equal amounts of protein were incubated at 37°C for 20 min in the presence or absence of 15 units of Endo H. Samples were kept on ice between each step.

### 2.7 B0AT1 protein Triton X-100 solubility assay

Eighteen hours post transfection, HEK293T cells cultured in 6 well-plates were harvested by collecting cells in media then centrifuged at 14,000 rpm for 20 min. The supernatant was discarded, and the pallet was washed with 500 μL of ice-cold 1x PBS (Phosphate buffer saline) followed by another centrifugation. The supernatant was discarded again, and the pellets were lysed with 80 µL of ice-cold 1x TBSt (Tris-buffered saline with 1%TritonX-100) and incubated on a rotary shaker at 4°C for 1 h thirty-5 μL of total sample were removed as the “total sample,” and remaining lysate was centrifuged at 14,000 rpm at 4°C for 20 min. The supernatant and pellets were collected in separate tubes. The pellet was resuspended in 50 µL TBSt and 22 μL of 4x Laemmli Sample Buffer (BioRad, Cat# 1610747) supplemented with 1.25% β-mercaptoethanol (BME). The pellet was sonicated at 25% power for three 13 s intervals. The “Supernatant” and “Total” samples for each condition were quantified using a BCA protein assay (Pierce, Cat# 23225). Thirty µg total protein lysates were resolved on a 10% SDS-PAGE gel, followed by semi-dry transfer to a PVDF membrane, as previously described in the SDS-PAGE immunoblotting method.

### 2.8 In silico prediction of B0AT1 mutations effects on the [ACE2-B0AT1] complex stability

Predicting protein complex stability of the [ACE2-B0AT1] complex with various B0AT1 mutations was performed using mCSM-PPI2, a novel machine learning method for predicting effects of missense mutations on protein-protein affinity. As described by the developers mCSM-PPI2, is an easy-to-use web server that implements an integrated computational approach for predicting effects of missense mutations in protein-protein affinity, this method uses graph-based structural signatures called mCSM which has been shown to be an accurate and high-throughput to predict the impact of mutations on protein structure and function, and was one of the first methods capable of assessing the impact of mutations on protein interaction binding affinity. The wild type complex used had PDB ID: 6M18. The stability change in a protein can be measured by calculating the change in its Gibbs-free energy upon folding. Substituting a single amino acid in a protein sequence can result in a significant change in protein stability (ΔΔG); positive ΔΔG value indicates a destabilizing effect, while a negative value indicates a stabilizing effect. The energy difference between native and mutant proteins was calculated based on Gibbs-free energy, and the predicted free energy change was denoted by ΔΔG value. We used the bioinformatics tool EMBL-EBI ProtVar for contextualising human missense variations. The scoring criteria were as follows: [*0.00–0.15* = very low conservation, *0.15–0.30* = low conservation, *0.30–0.45* = fairly low conservation, *0.45–0.60* = moderate conservation, *0.60–0.75* = fairly high conservation, *0.75–0.90* = high conservation, *0.90–1.00* = very high conservation]. PolyPhen-2 was used too for prediction of functional effects of human nsSNPs, http://genetics.bwh.harvard.edu/pph2/. PolyPhen-2 (Polymorphism Phenotyping v2) is a computational tool designed to assess the potential effects of amino acid substitutions on human protein structure and function. It employs fundamental physical and comparative analyses to generate predictions. Protein sequence and functional annotation data were retrieved from the UniProt database https://www.uniprot.org/, a comprehensive resource for protein information. UniProt was utilized to obtain details on sequence variations, functional domains, and relevant annotations to support the analysis of genetic variants. This dataset facilitated comparative assessments and structural insights, contributing to the interpretation of variant effects in our study.

## 3 Results

### 3.1 Exogenously expressed FLAG-Tagged wild type B0AT1 localizes to the plasma membrane but forms high molecular weight aggregates in SDS-PAGE analysis

**FIGURE 2:**
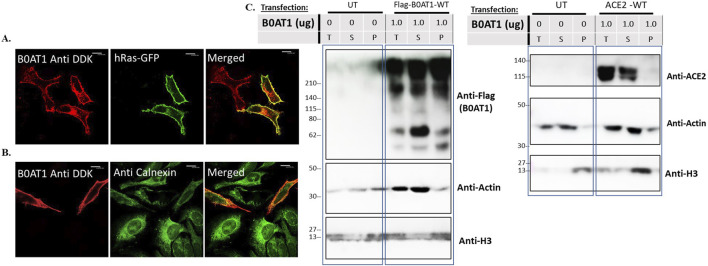
subcellular localization and behaviour of Wild type B0AT1 on SDS-PAGE. **(A,B)** The subcellular localization of B0AT1-WT was visualised in Hela cells transfected with FLAG-tagged B0AT1-WT, showing plasma membrane localization after 24 h of transfection. Co-localization with the plasma membrane marker GFP-hRas **(A)** and absence of co-localization with the ER marker Calnexin **(B)** confirm plasma membrane localization. **(C)** Solubility assay showing B0AT1 protein aggregates in lysates that partition into soluble (S) and the insoluble (P) fractions. Another blot showing ACE2 bands in the soluble (S) fraction. Actin was used as a loading control. The protein ladder used is BLUltra Pre-stained Protein Ladder (Cat# PM001-0500). Immunofluorescence images were captured with a Nikon eclipse i80 at magnification of ×100. Scale bar = 50 μm.

We aimed to confirm that wild type (WT) C-terminally FLAG-tagged B0AT1 localizes to the plasma membrane as expected. To achieve this, we expressed it from the mammalian expression plasmid (Origene #RC212948) in HeLa cells and performed immunofluorescence confocal microscopy imaging. B0AT1 appeared to predominantly localize to the cell periphery and co-localize with GFP-H-Ras, a plasma membrane marker, as evidenced by the yellow signal resulting from the overlap of the two images when merged (Figure 2A). On the other hand, as expected, it showed the majority of the protein transported to the plasma membrane with some co-localization with the ER marker, calnexin (Figure 2B). This fraction is presumably the fraction of the WT protein that is still in transit or that failed to attain the proper folding.

Preliminary SDS-PAGE analysis of expressed B0AT1 revealed aberrant behaviour, prompting further evaluation before analysing disease-causing mutations. Our initial observation indicated almost complete aggregation of B0AT1 when the protein was heated at high temperatures in sample buffer. We therefore tried to optimize B0AT1 solubilization for SDS-PAGE analysis with various approaches including reducing the temperature, varying the extraction buffer (RIPA vs. PBS) adding beta-mercaptoethanol, DDT, sonication of the lysates without much luck. However, a fraction of the protein appeared to be soluble and migrated to the expected molecular weight in the gels was achieved (see Supplementary Figure S2). Further optimization was carried which led to the final protocol using an extraction buffer designed for the extraction of transmembrane proteins (Fivephoton Biochemicals, model# tmPER-50TM), with 1.25% BME and no heat. B0AT1 is a highly hydrophobic protein due to its 12 transmembrane domains (Figure 1). Therefore, solubility assays were performed to assess whether the protein was predominantly fractionates into the soluble or insoluble fraction, alongside other highly hydrophobic proteins such as Histones (e.g., H3) as shown in Supplementary Figure S3. Since H3 appeared only in the total insoluble part, while Actin appeared exclusively in the soluble fraction, these proteins were used as controls. As shown in Figure 2C, B0AT1 appeared in both the soluble (S) and insoluble (P) fractions, but significantly exhibiting a noticeable smearing and possible aggregation at the top of the resolving portion of the SDS-PAGE gels. However, a small portion of the protein seem to resolve within the resolving gel, appearing as two protein bands in the 55–75 KDa range. Two bands formed because of the solubility assay processing although normal SDS PAGE runs show only one band at this molecular weight (Supplementary Figures S2, S4, S5). While B0AT1 was present in both the insoluble and the soluble fractions, it was predominantly found in the latter (Figure 2C). On the other hand, ACE2 appeared exclusively in the soluble fraction (Figure 2C). Collectively these results suggest that while B0AT1-WT localizes to the plasma membrane *in vitro*, its hydrophobic nature and tendency to aggregate is expected to complicate SDS-PAGE and other biophysical analyses.

### 3.2 Subcellular localization analysis of 18 Hartnup disease-causing variants reveal ER retention of nine variants

**FIGURE 3:**
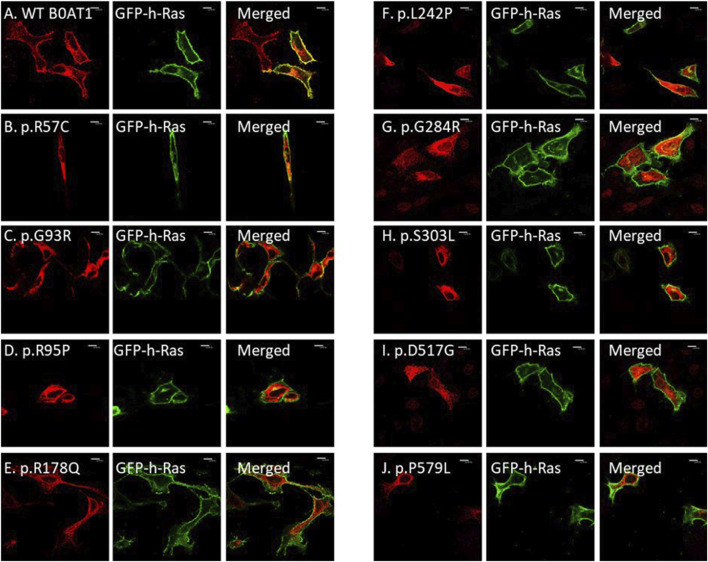
The 9 missense mutations do not co-localize with the plasma membrane marker. **(A)** B0AT1-WT shows plasma membrane localization. **(B–J)** Immuno-cytochemistry images of B0AT1 mutants (SLC6A19) localization; Immuno-cytochemistry images of B0AT1 with single nucleotide generated via site directed mutagenesis. These mutants show no normal localization, without co-localization with the PM marker GFP-hRas. Immunofluorescence images were captured with a Nikon eclipse i80 at magnification of ×100. Scale bar = 50 μm.

**FIGURE 4:**
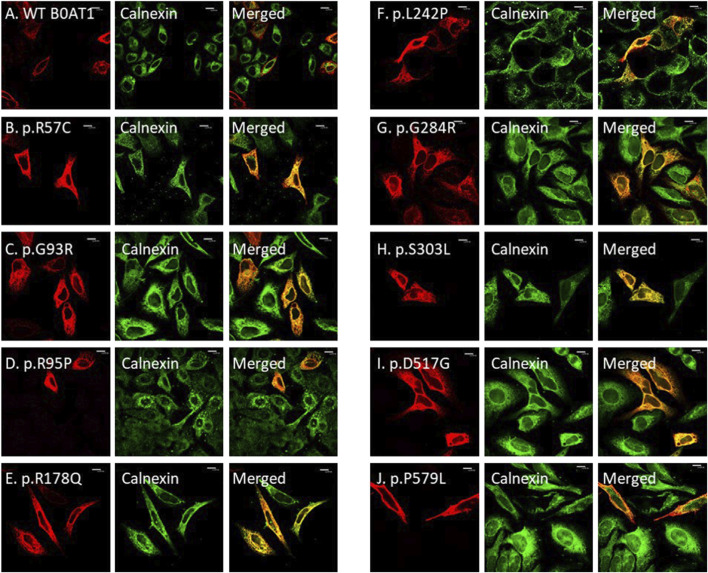
The 9 missense mutations co-localize with the ER marker. **(A)** B0AT1-WT shows plasma membrane localization with no ER retention. **(B–J)** Immuno-cytochemistry images of B0AT1 mutants (SLC6A19) generated via site-directed mutagenesis. The mutants show ER retention, confirmed by co-localization with Calnexin (an ER marker), while H-Ras serves as the plasma membrane marker. Immunofluorescence images were captured with a Nikon eclipse i80 at magnification of ×100. Scale bar = 50 μm.

We wanted to investigate the effects of several missense mutations associated with Hartnup disease, which affects various domains of B0AT1, on its subcellular localization and trafficking in comparison with wild type. As shown in Figures 2A,B, WT B0AT1 predominantly localized to the plasma membrane, serving as a qualitative indicator of proper trafficking and maturation. Co-localization analysis confirmed that WT B0AT1 overlapped with GFP-H-Ras (a plasma membrane marker), producing a yellow signal in merged images (Figure 3A), with a small fraction clearly co-localizing the ER marker, calnexin (Figure 4A).

Confocal immunofluorescence microscopy revealed that 9 (R57C, G93R, R95P, R178Q, L242P, G284R, S303L, D517G, P579L) of the 18 tested missense mutations (Supplementary Figure S1) seem to deviate significantly from the plasma membrane localization observed for the WT protein. These mutants showed minimal or no co-localization with the plasma membrane (GFP-Ras) marker (Figures 3B–J) but extensive co-localization with the ER marker, calnexin (Figures 4B–J). However, the remaining nine variants (G66R, A69T, P154L, S169T, D173N, R240Q, P265L, R328C, E405K) exhibited largely a plasma membrane subcellular localization similar to that of the WT protein (Supplementary Figure S1).

### 3.3 Colocalization analysis confirms plasma membrane localization of nine B0AT1 variants

Colocalization analysis was performed using CellProfiler to quantitatively assess the spatial relationship between the B0AT1 signal (red) and reference markers for the endoplasmic reticulum (blue) and the plasma membrane (green). Multiple colocalization metrics were computed, including Pearsons’ correlation, Manders’ coefficients, RWC coefficient and Slope to robustly determine the subcellular localization of B0AT1. The Pearsons’ correlation coefficient between red and green signals was 0.971, markedly higher than that between red and blue (0.343), indicating stronger colocalization with the plasma membrane marker. This observation was consistently supported by additional parameters:

• Manders’ Coefficient (red-green): 0.694/0.895. RWC Coefficient (red-green): 0.694/0.895. Slope (red-green): 0.943.

In contrast, colocalization metrics for red and blue were significantly lower:

• Manders’ Coefficient (red-blue): 0.017/0.008. RWC Coefficient (red-blue): 0.017/0.008. Slope (red-blue): 0.330.

Collectively, these results confirm that wild type B0AT1 predominantly colocalizes with the plasma membrane marker (green) and not with the ER marker (blue), supporting its plasma membranal localization under the experimental conditions. Similarly, this standardized pipeline was applied uniformly across Plasma membranal B0AT1 Variants, and confirmed their localization computationally (Supplementary Table S2).

### 3.4 N-glycosylation profiling reveals ER retention and maturation defects in disease-causing B0AT1 variants

We wanted to use Endoglycosidase H (Endo H) digestion assays to further confirm the ER retention of disease-causing variants. However, the aberrant behaviour of the WT protein on SDS-PAGE presented technical challenges for this analysis. The WT protein exhibited a significant smear and a distinct protein band around the expected size range of 55–75 kDa which represent the soluble fraction of the protein, on the other hand majority of the protein appears within the insoluble fraction as a smear and at the top of the resolving gel. Although Wild type protein digestion with Endoglycosidase F (PNGase F) showed a clear band shift yet, endo H digestion did not produce a band shift as shown in (Supplementary Figure S4). This analysis was difficult for the mutants, looking at the soluble fraction of the protein that resolved through the gel, we observed a single band that was digestible with Endo H (Supplementary Figure S5), corresponds to the immature suggesting it was in its immature state (ER-resident). Among the tested variants (Supplementary Figure S5), 10 (A69T, G93R, R95P, P154L, S169T, R178Q, R240Q, L242P, P265L, G284R) displayed a single band within the 55–75 kDa size range in the resolving gel, which was completely cleaved (showing a mobility shift) after Endo H treatment. This indicates that these proteins are immature, as evident by incomplete glycosylation and susceptibility to digestion. The R178Q variant exhibited two bands, suggesting that a fraction of the protein may undergo partial maturation. This is well documented in the literature and we have shown this to be the case for many proteins that we have previously studied (Alkhofash and Ali, 2024; Badawi et al., 2022; Gariballa and Ali, 2020; Hume et al., 2009; Jawabri et al., 2024; Kizhakkedath et al., 2019). These findings align with the fluorescence microscopy results, further confirming the extensive ER retention of these nine mutants, either completely or partially. Although, due to the presence of the presumed aggregates at the top of the resolving gel, this interpretation should be considered as preliminary because of the unknown glycosylation status of these aggregates.

Although the Endo H digestion assay results are complicated by the aberrant behavior of the B0AT1 protein on SDS-PAGE gels, they are largely consistent with immunofluorescence imaging, thus confirming the ER retention of at least nine of the tested mutants. This suggests that aggregation may interfere with electrophoretic separation, warranting further experimental conditions optimization.

### 3.5 B0AT1 variants influence on WT ACE2 trafficking and maturation when co-expressed

#### 3.5.1 Co expression of ER-retained B0AT1 variants influence the subcellular localization of WT ACE2

**FIGURE 5:**
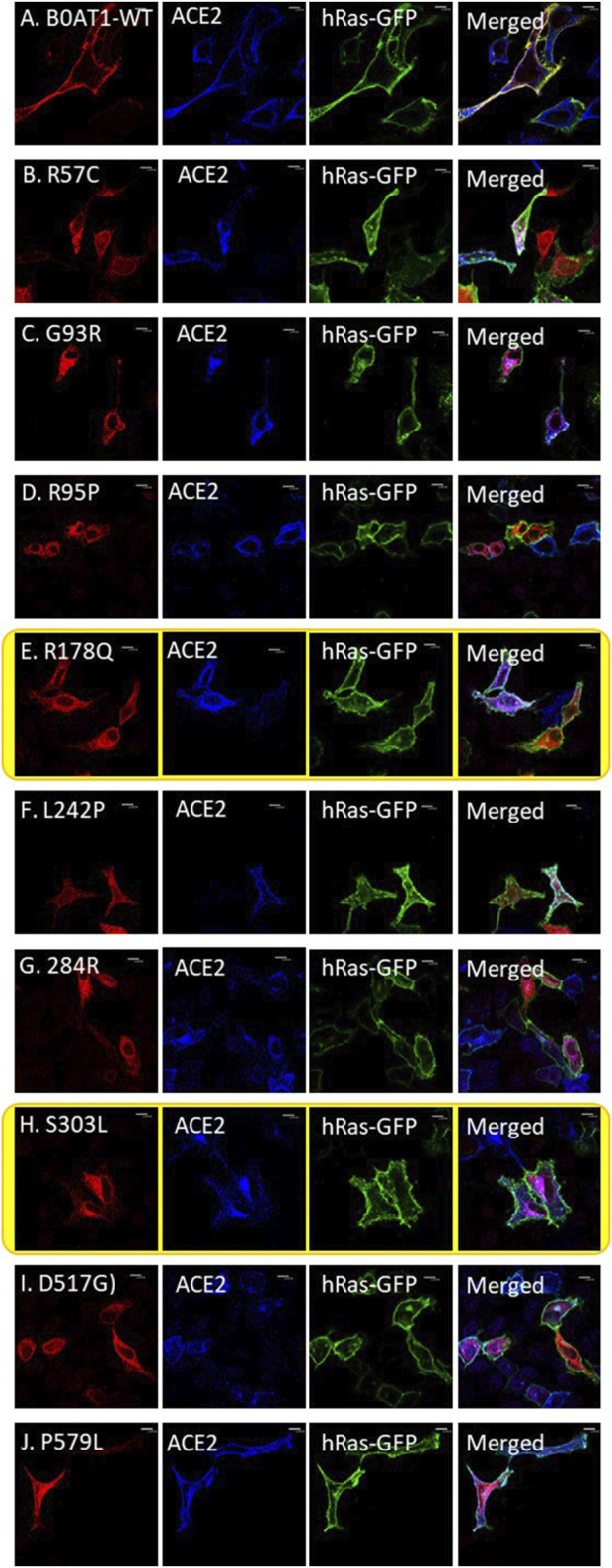
ER-retained B0AT1 variants and ACE2 co-transfection with GFP-hRas in Hela cells. **(A)** Co-localization of WT-ACE2 (Blue) with the plasma membrane marker GFP-hRas (Green). **(B–J)** Co-localization of ER-retained B0AT1 (SLC6A19) mutants (Red) and WT-ACE2 (Blue) Immuno-cytochemistry show the of B0AT1 mutants on ACE2 localization. Immunofluorescence images were captured with a Nikon eclipse i80 at magnification of ×100. Scale bar = 50 μm.

To investigate the potential effects of ER-retained missense mutations in B0AT1 on ACE2 subcellular localization, we first assessed the impact of WT B0AT1 on co-expressed WT ACE2. As expected, WT ACE2 exhibited normal plasma membrane trafficking and extensive co-localization with GFP-HRas and B0AT1, as shown in Figure 5A.

**FIGURE 6:**
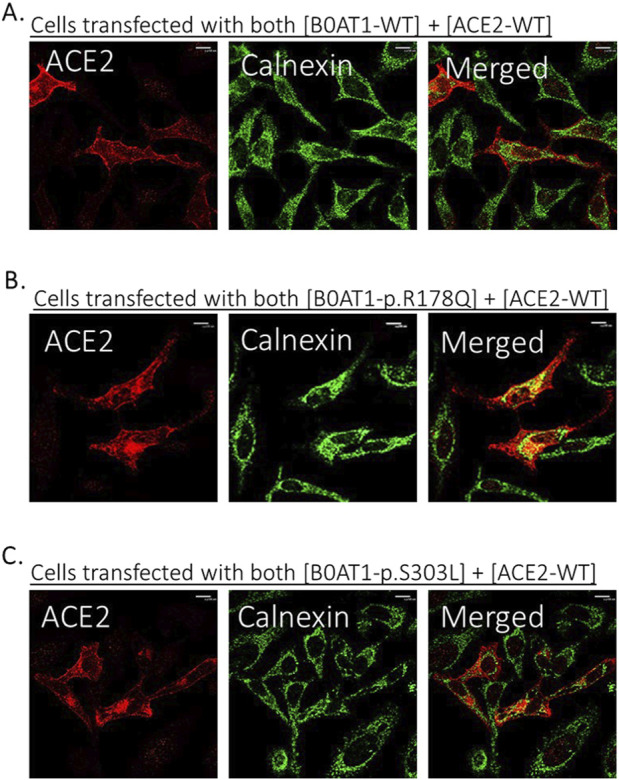
Co-expression of B0AT1 and ACE2 affects ACE2 localization. Hela cells were transfected with Flag-tagged B0AT1 and untagged ACE2. Cells were harvested 18 h post-transfection. **(A)** WT B0AT1 co-localizes with ACE2 at the plasma membrane. **(B,C)** ER-retained B0AT1 variants co-localize with ACE2 in the ER. Anti-FLAG antibody (mouse) was used to label B0AT1 in red, anti-ACE2 antibody (rabbit) to label ACE2 in blue, and anti-Calnexin antibody (goat) to label the ER marker in green. Immunofluorescence images were captured using a Nikon Eclipse i80 microscope at ×100 magnification. Scale bar = 50 μm.

However, two of the ER-retained B0AT1 missense mutants (R178Q and S303L) seemed to disrupt the subcellular localization of co-expressed ACE2, preventing its successful transport to the plasma membrane but instead ACE2 seemed to be retained intracellularly (Figures 5E,H). This was further confirmed by co-localization with the ER marker calnexin (Figure 6). Notably, R178Q in (Figure 6A) showed slightly more co-localization of co-expressed ACE2 with Calnexin (ER marker) than S303L (Figure 6B).

#### 3.5.2 Co-expression of ER-retained B0AT1 mutants resulted in ACE2 aggregation, as analysed by SDS-PAGE

We aimed to further explore the impact of the two ER-retained B0AT1 mutants (R178Q and S303L) on WT ACE2 trafficking.

**FIGURE 7:**
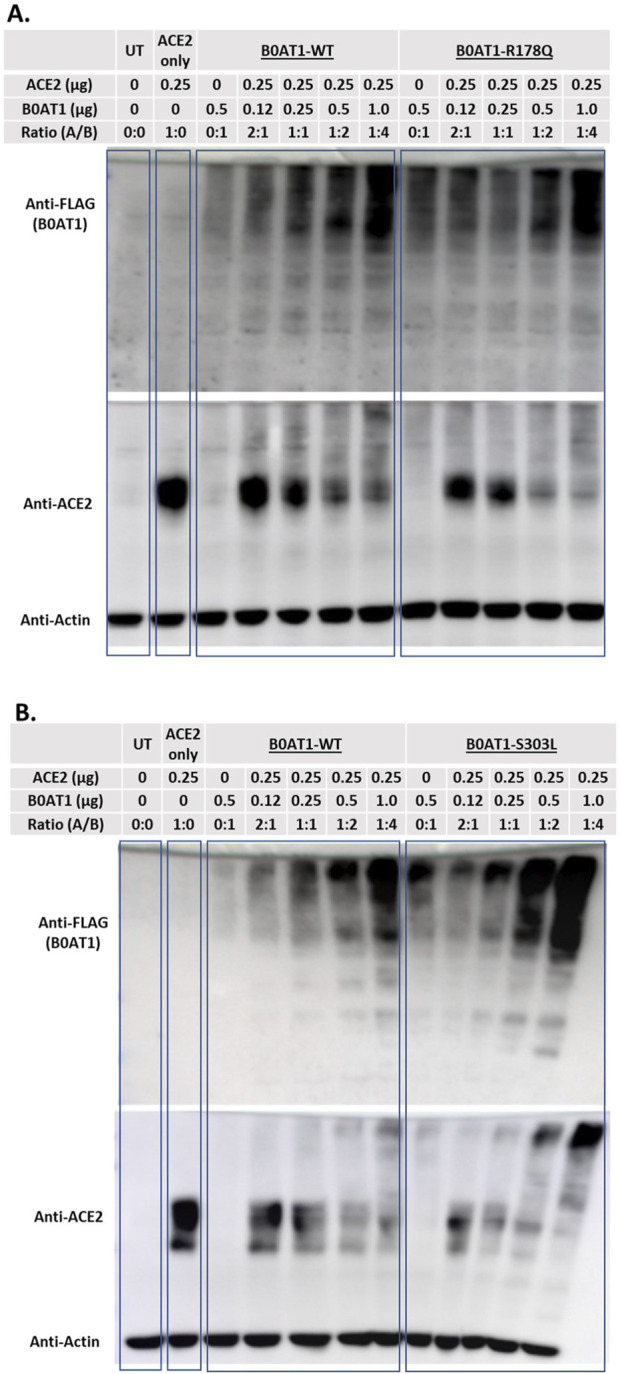
ACE2 forms complexes with B0AT1. Hek293T cells were co-transfected with FLAG-tagged B0AT1 (wild type or variants) and untagged ACE2. **(A)** Comparison of WT B0AT1 and R178Q in the SDS-PAGE assay, showing ACE2 aggregation. **(B)** Same as panel A but with the S303L mutant. A blot was stripped of anti-FLAG and re-probed with anti-ACE2, followed by anti-actin as a loading control. The UT stands for un-transfected cells lysate. Un-Transfected (UT) as an experimental control. The protein ladder used is the Precision Plus Dual Color Protein Ladder (Cat# 1610374).

To confirm the interaction of WT ACE2 with BOAT1, including the two ER-retained mutants, and to further evaluate the impact of these mutants on ACE2 subcellular localization (Figures 5,6), cells were co-transfected with the selected plasmids in varying DNA:DNA ratios. The effect of B0AT1 on ACE2 was then examined by analysing ACE2 in SDS-PAGE gels (Figure 7). The results revealed a decrease in the usual protein band intensity of ACE2 at the 120 kDa position as the amount of expressed B0AT1 (WT and the two ER-retained mutants, R178Q and S303L) increased. Concomitantly, there was an increase in aggregate formation at the top of the resolving gel, along with smearing within the gel. This suggests that, as B0AT1 levels rise, more ACE2-B0AT1 super-complexes are formed, which then aggregate, likely due to B0AT1’s hydrophobicity (Figures 7A,B). These results demonstrate a decrease in WT ACE2 band intensity at 120 kDa position. In addition, they demonstrate an increase in the smear at higher molecular weights, indicating protein aggregation, likely with B0AT1, confirming the ACE2-B0AT complex formation and subsequent aggregation during sample processing.

**FIGURE 8:**
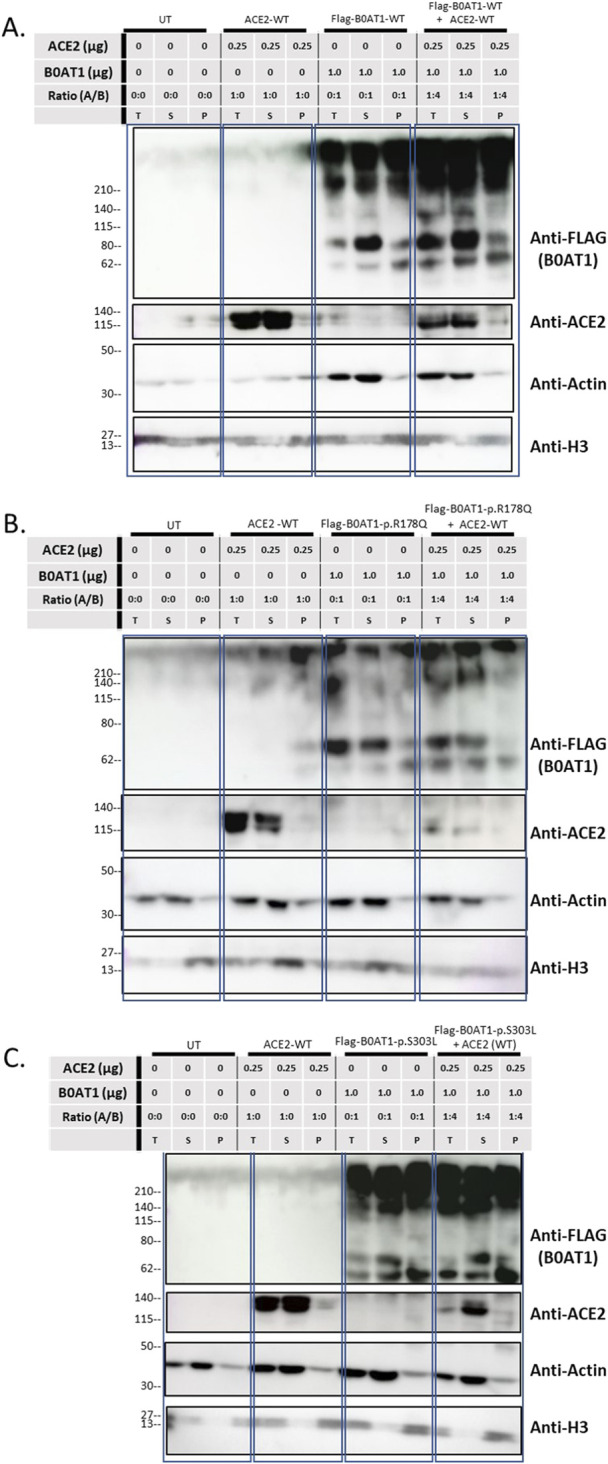
Solubility assay reveals ACE2 complexes with B0AT1. Wild type ACE2 and FLAG-tagged B0AT1 were co-transfected into Hek293T cells, followed by segregation via solubility assay through centrifugation. **(A)** Co-transfection of WT ACE2 and B0AT1-WT. **(B)** Co-transfection of WT ACE2 with the ER-retained B0AT1 variant R178Q. **(C)** Co-transfection of WT ACE2 with the ER-retained B0AT1 variant S303L. Samples were separated into total (T), supernatant (S), and pellet (P) fractions. Anti-FLAG labels B0AT1, and anti-ACE2 labels ACE2. H3 is used as separation control (see Supplementary Figure S3). Actin is used for separation and loading control. The UT stands for un-transfected cells lysate. Un-Transfected (UT) as an experimental control. Protein ladder used is the BLUltra Pre-Stained Protein ladder (Cat# PM001-0500).

To investigate whether these aggregates were present in the insoluble fraction, solubility assays were conducted by dividing the lysate into soluble (S) and insoluble (P) phases, with “T” representing the total sample (Figure 8). When B0AT1-WT was co-expressed with ACE2-WT, heavier bands aggregated at the top of the blots across all phases of the cell lysate, as detected by anti-FLAG. However, bands corresponding to smaller molecular sizes were predominantly observed in the soluble phase, with fewer in the insoluble pellet phase (Figure 8A). The same experiment was repeated with the R178Q and S303L variants to examine the effect of ACE2-B0AT1 complex formation on ACE2 solubility. The results showed that ACE2 bands were predominantly in the soluble phase but decrease in intensity when co-expressed with each variant (Figures 8B,C). Compared to ACE2 alone, almost all ACE2 was bound to B0AT1, forming aggregates at the top of the blot and disappearing from the 120 kDa region when co-expressed with R178Q (Figure 8B) and S303L (Figure 8C).

### 3.6 *In silico* analysis of the effect of B0AT1 missense variants on ACE2 interaction

Protein-protein interactions play a crucial role in mediating most cellular processes, and it is well established that disease-causing mutations are often enriched at these interfaces. In recent years, various computational approaches have been proposed to study mutation impact on protein complexes. In this study, we used mCSM-PPI2, a novel machine learning method, to predict missense mutation effects on protein-protein affinity. As described by the developers of the software this approach employs an optimised graph-based structural signatures called mCSM to assess molecular mechanisms of mutations more effectively. It models the impact of variations on the inter-residue non-covalent interaction network using graph kernels, evolutionary information, complex network metrics, and energetic terms.

Our focus was on the ER-retained variants and their effects on protein stability, localization, functionality, and ACE2 binding affinity. Among the nine ER-retained variants, R95P and S303L showed “high conservation,” while the remaining seven variants were predicted to have “very high conservation,” indicating that their sequences are preserved throughout the solute carrier 6 (SLC6) family, and alterations would likely affect function.

#### 3.6.1 SLC6A19 c.169C>T (Arg57Cys)

**FIGURE 9:**
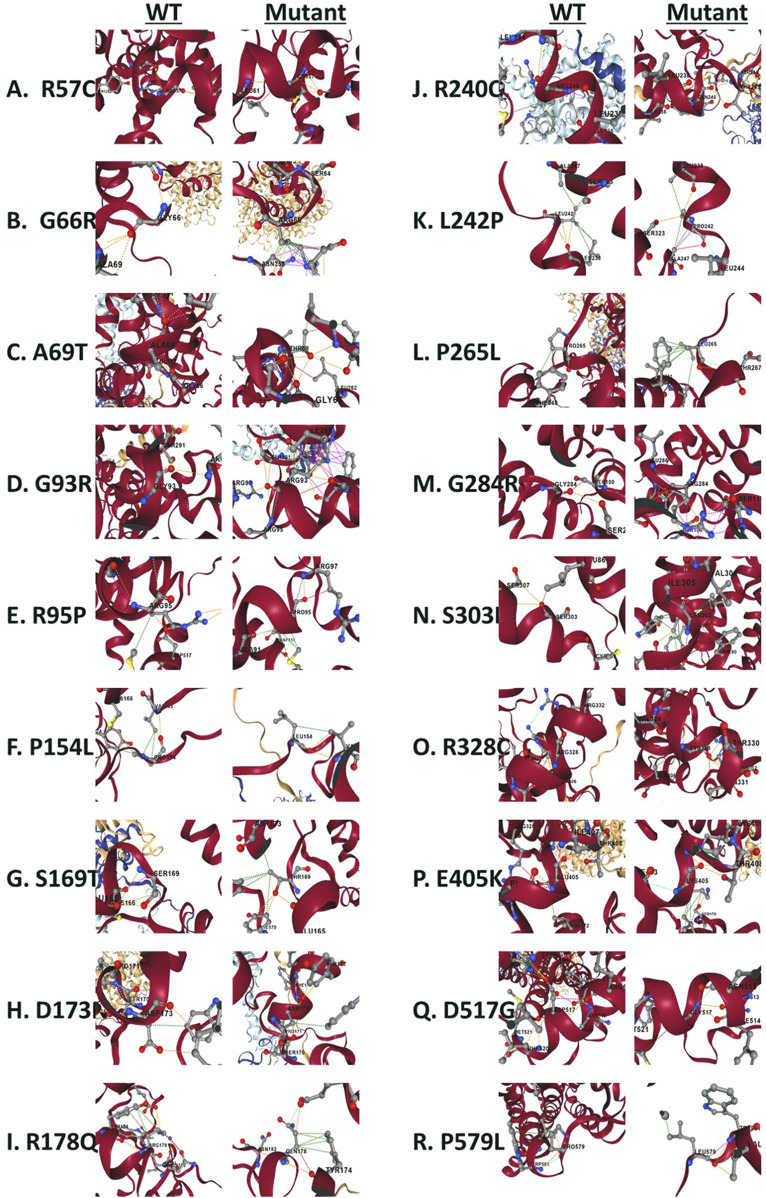
Structural view of residues surrounding missense variants. **(A)** Wild type Arg57 vs. mutant Cys57. **(B)** wild type Gly66 vs. mutant Arg66. **(C)** wild type Ala69 vs. mutant Ther69. **(D)** wild type Gly93 vs. mutant Arg93. **(E)** wild type Arg95 vs. mutant Pro95. **(F)** wild type Pro154 vs. mutant Lue154. **(G)** wild type Ser169 vs. mutant Thr169. **(H)** wild type Asp173 vs. mutant Asn173. **(I)** wild type Arg178 vs. mutant Gln178. **(J)** wild type Arg240 vs. mutant Gln240. **(K)** wild type Leu242 vs. mutant Pro242. **(L)** wild type Pro265 vs. mutant Lue265. **(M)** wild type Gly284 vs. mutant Arg284. **(N)** wild type Ser303 vs. mutant Leu303. **(O)** wild type Arg328 vs. mutant Cys328. **(P)** wild type Glu405 vs. mutant Lys405. **(Q)** wild type Asp517 vs. mutant Gly517. **(R)** wild type Pro579 vs. mutant Lue579. The wild type complex (PDB ID: 6M18) is shown in a cartoon representation. B0AT1 is represented by Chains A (red) and C (gray), while ACE2 consists of chains B and chain D (yellow). Amino acid residues are depicted as sticks.

This variant was ER-retained (Figures 3, 4) but does not affect ACE2 localization (Figure 5). The variant replaces arginine (basic and polar) with cysteine (neutral and slightly polar) at codon 57. In-silico tools predict a decreased affinity for the ACE2-B0AT complex (Figure 9; Table 1). Literature reports this variant’s deleterious and associated with Hartnup Disease (Kleta et al., 2004; Seow et al., 2004). Experimental evidence (Camargo et al., 2009; Kleta et al., 2004; Seow et al., 2004) shows no transport activity when compared to wild type, either when expressed alone or co-expressed with ACE2, leading to the loss of surface display and ER retention (Figure 5). Invitae Labs (2024) reports advanced modelling indicates this missense variant disrupts SLC6A19 protein function with a predictive value of 80%. However, it is classified as a “Variant of Uncertain Significance” due to insufficient evidence. Loss-of-function variants in SLC6A19 are known to be pathogenic (Kleta et al., 2004; Seow et al., 2004). This variant (R57C) is classified by UniPort as deleterious (Uniprot ID: VAR_023314) and by ClinVar as likely pathogenic in 2023, updated to Uncertain Significance in 2024 (ClinVar ID: VCV002503943.2).

**TABLE 1:** *[ACE2-B0AT1] complex* stability profile and energy calculations upon B0AT1 variants involvement.

Serial#	SNP	HGVS_cDNA	Location	HGVS_protein	Stability	ΔΔG (Kcal/mol)
01	rs762989809	NM_001003841.3:c.169C>T	Helical,1	NP_001003841.1:p.R57C	Decrease	−0.326
02	rs1251095994	NM_001003841.3:c.196G>A	Extracellular	NP_001003841.1:p.G66R	Decrease	−0.518
03	rs1745926443	NM_001003841.3:c.205G>A	helical,2	NP_001003841.1:p.A69T	Increase	0.184
04	rs757679627	NM_001003841.3:c.277G>A	cytoplsmic	NP_001003841.1:p.G93R	Decrease	−0.495
05	rs201936518	NM_001003841.3:c.284G>C	cytoplasmic	NP_001003841.1:p.R95P	Decrease	−0.956
06	rs771980756	NM_001003841.3:c.461C>T	extracellular	NP_001003841.1:p.P154L	Decrease	−0.342
07	Na	NM_001003841.3:c.506G>C	extracellular	NP_001003841.1:p.S169T	Decrease	−0.034
08	rs121434346	NM_001003841.3:c.517G>A	extracellular	NP_001003841.1:p.D173N	Decrease	−0.542
09	rs763090941	NM_001003841.3:c.533G>A	extracellular	NP_001003841.1:p.R178Q	Decrease	−0.138
10	rs758492838	NM_001003841.3:c.719G>A	helical,5	NP_001003841.1:p.R240Q	Decrease	−0.332
11	rs200745023	NM_001003841.3:c.725T>C	Helical,5	NP_001003841.1:p.L242P	Decrease	−1.007
12	rs148139045	NM_001003841.3:c.794C>T	extracellular	NP_001003841.1:p.P265L	Decrease	−0.009
13	rs200842846	NM_001003841.3:c.850G>A	Helical,6	NP_001003841.1:p.G284R	Decrease	−0.743
14	rs201212000	NM_001003841.3:c.908C>T	cytoplasmic	NP_001003841.1:p.S303L	Decrease	−0.132
15	rs142164435	NM_001003841.3:c.982C>T	extracellular	NP_001003841.1:p.R328C	Decrease	−0.09
16	rs765501634	NM_001003841.3:c.1213G>A	cytoplsmic	NP_001003841.1:p.E405K	Decrease	−0.192
17	rs745524993	NM_001003841.3:c.1550A>G	cytoplasmic	NP_001003841.1:p.D517G	Decrease	−0.097
18	rs751554174	NM_001003841.3:c.1736C>T	extracellular	NP_001003841.1:p.P579L	Decrease	−0.034

SNP, single nucleotide polymorphism; HGVS, human genome variation society nomenclature; AA, amino acid; ΔΔG, gibbs free energy.

#### 3.6.2 SLC6A19 c.284G>C (Arg95Pro)

This variant shows high conservation and is predicted to be unlikely to destabilize the protein, yet it shows decreased affinity for ACE2-B0AT1 binding (Table 1). It does not have a ClinVar entry but was reported as “likely pathogenic” in Ensembl (Ensembl ID: rs201936518) as part of the 10,000 Genomes project (Ensembl, 2020) and is predicted by PolyPhen to be “probably damaging” (0.963).

#### 3.6.3 SLC6A19 c.850G>A (Gly284Arg)

This variant does not have a ClinVar entry but is classified as pathogenic in UniProt (VAR_081075) and is associated with Hartnup disease. Functional studies show that Gly284Arg abolishes amino acid transport activity (Azmanov et al., 2008). Using a publicly available web tool (https://www.ebi.ac.uk/ProtVar), we found that two mutation cause Gly/Arg missense mutation; one of them is our assayed (G>A) and the other is (G>C). Both variants are highly conserved and predicted to destabilize the protein. However, the G>C mutation has a lower score for predicted pathogenicity (29.7, “probably deleterious”), while (G>A) has a higher score (32, “highly likely deleterious”) according to CADD (PMID: 30371827).

#### 3.6.4 SLC6A19 c.1550A>G (Asp517Gly)

Prediction tools find this variant is likely destabilizing, but it only slightly decreases ACD2-B0AT1 binding affinity (Table 1). This variant has no ClinVar entry but was experimentally proven by Azmanov and colleagues to be associated with Hartnup and functionally disrupt amino acid transport activity (Azmanov et al., 2008). It is classified as “Pathogenic” in UniProt (UniProt ID: VAR_081078).

#### 3.6.5 SLC6A19 c.1736C>T (Pro579Leu)

This variant does not have a ClinVar entry and is predicted to be likely destabilizing, decreasing ACD2-B0AT1 binding affinity (Table 1). Experimental studies (Camargo et al., 2009) show no amino acid transport activity and loss of surface expression when expressed alone or co-expressed with ACE2. This variant has no ClinVar entry. The C>T mutation is likely to be destabilizing and quite likely deleterious according to CADD. Another mutation (C>G) shows same predictions and has a lower allele frequency in the population, according to GenomaD.

#### 3.6.6 SLC6A19 c.533G>A (Arg178Gln)

This variant shows very high conservation and is predicted to be unlikely to destabilising the protein, yet it shows decreased affinity for ACE2-B0AT1 binding (Table 1). Arg178Gln does not have a ClinVar entry and its function is unknown, but its pathogenicity is predicted to be highly likely deleterious (using *in silico* tool PolyPhen). We observed that ACE2 was retained in the ER when co-expressed with this variant (Figures 5, 6B).

#### 3.6.7 SLC6A19 c.908C>T (Ser303Leu)

This is an ER-retained variant that causes ACE2 ER retention when co-expressed (Figures 5, 6C). The sequence change replaces serine (neutral and polar) with leucine (neutral and non-polar) at codon 303. ClinVar reports it as a neutral amino acid transport defect (ClinVar ID: VCV000916043). In-silico predictions suggest it is “highly destabilizing” with decreased affinity for the ACE2-B0AT1 complex (Table 1). (Cheon et al., 2010) identified this mutation as somatic in a Hartnup patient (Cheon et al., 2010). This variant is classified as a “Variant with uncertain significance” in both ClinVar and Uniprot, according to ACMG/AMP guidelines (Nykamp et al., 2017). Invitae Labs also reports that this missense variant is not expected to disrupt SLC6A19 protein function, PolyPhen predicts it to be “benign (0.24)”.

## 4 Discussion

This article evaluates the subcellular localization of 18 Hartnup disease-causing B0AT1 variants and assesses their impact on the subcellular localization of WT ACE2 when co-expressed in cultured cell lines (HEK 293T and HeLa). We selected HEK 293T cells as a model because they are a human embryonic kidney cell line that lacks the endogenous expression of B0AT1, as indicated by the Human Protein Atlas (Cell Line - SLC6A19 - The Human Protein Atlas, 2024; Gonçalves et al., 2022). Both HEK 293T and HeLa express high levels of a aminopeptidase N (CD13), a protein reported to enhance B0AT1 expression in the intestinal lumen, encoded by the gene NUP210 (Fairweather et al., 2012).

Mutations in the *SLC6A19* gene cause to malfunctioning of this transporter, leading to Hartnup disorder, an autosomal recessive condition characterized by urinary loss of neutral amino acids due to an inability to transport them across the cellular brush border (Kleta et al., 2004; Seow et al., 2004). However, it remains uncertain whether these variants affect the protein sub-cellular localization. Specifically, we hypothesized that some of these single nucleotide mutations may cause the protein to remain within intracellularly, away from the plasma membrane, which is the normal site for B0AT1-WT sodium dependent amino acid transport activity.

B0AT1 has 5 potential N-glycosylation sites (158, 182, 258, 354, 368) across its sequence as indicated in the diagram presented in Figure 1. The immature form of the protein is the high-mannose glycosylated one that resides in the ER, and thus should be sensitive to endo-H. The mature form is complex-glycosylated, making it resistant to endo-H and sensitive only to Endoglycosidase F (PNGase F). Similar to wild type B0AT1, upon their transport to the Golgi and acquiring further modifications to their N-glycans, they become endo H resistant. This reflects the complete maturation of the protein and successful transport to the plasma membrane. In Supplementary Figure S4 we see the endo H resistant wild type is digested by PNGase F because both forms of the N-glycans are cleavable with this enzyme. This phenomenon is thoroughly documented in the literature, and our previous work has demonstrated it for numerous proteins (Badawi et al., 2022; Gariballa and Ali, 2020; Hume et al., 2009; Jawabri et al., 2024; Kizhakkedath et al., 2019). The ER located variants are expected to be sensitive to digestion by Endo H, for example mutant R178Q in Supplementary Figure S5, shows a clear band shift that confirms proteins’ inability to mature and reach the plasma membrane which calls for Endoplasmic-reticulum-associated protein degradation (ERAD) machinery to eradicate ER stuck proteins. Our lab and other international labs have successfully tracked ERAD machinery function and their significant role in health and disease (Gariballa et al., 2024; Jawabri et al., 2024; Kizhakkedath et al., 2019; Meusser et al., 2005; Needham et al., 2019; Tepedelen et al., 2019).

Our experiments revealed that 9 of the 18 evaluated mutants resulted in ER retention of B0AT1 (Figures 3, 4). Additionally, two of these mutants affected subcellular localization of WT ACE2, likely leading to its ER retention (Figure 5;Supplementary Figure S3). Only one variant showed increased ACE2-B0AT1 binding affinity, as predicted by *in silico* analysis (Figure 9; Table 1), and had a plasma membrane localization, allowing for normal cell surface display.

The p.A69T mutant was the only variant among the 18 studied to show “increased” ACE2-B0AT1 binding affinity, as predicted by *in silico* tool (Figure 9; Table 1). Literature suggests this variant has high structural stability. Our localization assay (Figures 3,4,6) is consistent with previous findings, such as those by Camargo et al. (2009), who reported that this Hartnup-associated variant increases cell membrane localization in the presence of ACE2 without affecting its interaction with ACE2. However, functional assays showed that the A96T variant abolished amino acid transport activity when co-expressed with ACE2 (Camargo et al., 2009).

Based on *in silico* prediction tools, we discuss the impact of ER-retained B0AT1 on cellular physiology, its association with disease phenotypes, and the potential for therapeutic targeting. Further studies are required to investigate the effects of B0AT1 variants on their expression, function, and binding to accessory proteins.

Mutants exhibiting ER retention affected ACE2 localization to varying degrees as indicated by co-localization with both B0AT in the ER and hRras in the plasma membrane. However, two missense mutants (R178Q and S303L) caused ACE2 to be retained in the ER, preventing its co-localization with the plasma membrane marker and instead co-localizing exclusively with ER-retained B0AT1 (Figures 5, 6).

Literature emphasizes the connection between Hartnup disorder and the cardiovascular system. A meta-analysis included 13 studies involving 34,370 individuals concluded that decreased circulating tryptophan levels are associated with an increased risk of CVD. Interventions to modulate tryptophan levels may prevent CVD (Zhang et al., 2024). Pharmacological agents targeting ACE2 could enhance its cardiovascular protective roles while affecting B0AT1 functionality. Recent approaches involving ACE2 agonists have shown promise in preclinical trials (El-Aziz et al., 2020; Gasperetti et al., 2022; Zhang et al., 2021). Such therapies could offer effective treatments of diseases linked to both proteins.

While the absence of B0AT1 causes Hartnup disease, it has also been associated with improved glucose tolerance (Garrido et al., 2022; Jiang et al., 2015), and reduced liver triglycerides (Yadav et al., 2020). B0AT1 has also been shown to lower elevated amino acid levels in mouse models of phenylketonuria and urea cycle disorders (Belanger et al., 2018; Seow et al., 2004) and may protect against kidney damage (Garrido et al., 2022). Consequently, B0AT1 inhibitors are being studied for their therapeutic potential (Cheng et al., 2017; Pochini et al., 2014; Scalise and Indiveri, 2020; Xu et al., 2024).

To discuss the limitations of our study we must say that the highly hydrophobic nature of B0AT1 led to difficulties in protein resolving through the SDS-PAGE gel and this created biochemical assay issues. Under heat, B0AT1 appeared as aggregates of FLAG-tagged proteins at the top of SDS-PAGE gels. Optimizations were considered to try and over-come this limitation, including using an extraction buffer designed for transmembrane proteins (Fivephoton Biochemicals, model# tmPER-50TM), and reducing methanol to 15% in SDS-PAGE transfer buffer. These conditions were used to better solubilize B0AT1 protein to resolve trough the gel for better visualization of its bands. All experiments were performed on ice, which may have affected lysates resolution due to skipped denaturation steps in biochemical assays.

A second limitation of our study is the need for a confirmatory test. Although we clearly show that B0AT1 colocalizes with both plasma membrane proteins hRas and ACE2-WT, yet complementary and specific approaches, such as cell surface biotinylation followed by western blotting, are required to better assess the plasma membrane expression of B0AT1 protein.

Another limitation is the cell model used. HEK293T and HeLa cell lines, may not fully represent other cell types or tissues. The HEK 293T cell line, in particular, could interfere with functional assay readings because it expresses two neutral amino acid transporters (ASCT2 and SNAT2), but their effects could be eliminated with specific inhibitory drugs.

Future research should investigate the effects of B0AT1 inhibitors on ACE2 and their potential to protect against ACE2-related diseases. Advanced modelling of protein sequences and biophysical properties such as structural, functional, and spatial information, amino acid conservation, physicochemical variation, residue mobility, and thermodynamic stability should be conducted to clarify the effect of each missense mutation.

In conclusion, the neutral amino acid transporter B0AT1, with Hartnup disease-associated mutations, has been experimentally shown to exhibit ER retention, suggesting the involvement of ERAD pathway in its pathogenesis (Christianson et al., 2011; Gariballa and Ali, 2020; Gariballa et al., 2024; Meusser et al., 2005; Qi et al., 2017; Tepedelen et al., 2019; Zacchi et al., 2022). Additionally, some ER-retained mutants affect WT ACE2 localization to varying degrees. Two missense mutants (R178Q and S303L) prevented ACE2 transport, trapping it in the intercellular compartment and hindering its reach to the plasma membrane, its site of functional activity. Future research should explore the functional activity and potential rescue strategies for ACE2, as well as evaluate the clinical implications for Hartnup patients carrying these variants.

## Data Availability

The original contributions presented in the study are included in the article/Supplementary Material, further inquiries can be directed to the corresponding author.
