# Supplementary material for: Hartnup disease-causing SLC6A19 mutations lead to B0AT1 aberrant trafficking and ACE2 mis-localisation implicating the endoplasmic reticulum protein quality control
Source: Front Cell Dev Biol. 2025 Aug 7;13:1589534. doi: 10.3389/fcell.2025.1589534 (PMC12368292; doi:10.3389/fcell.2025.1589534)
Supplement: Supplementary file 1 [file Supplementaryfile1.pptx]

## Slide 1
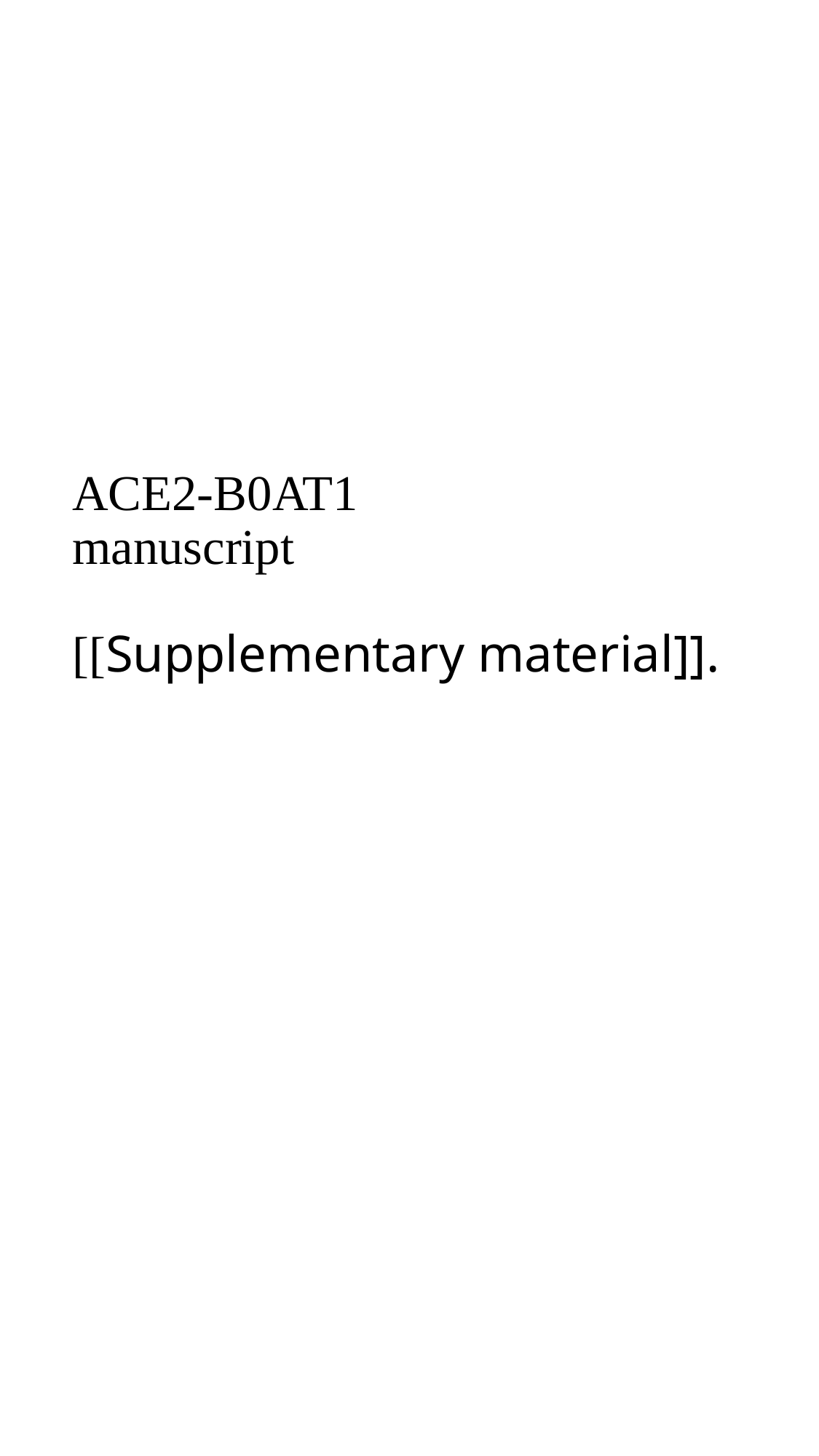

ACE2-B0AT1
manuscript
[[Supplementary material]].

## Slide 2
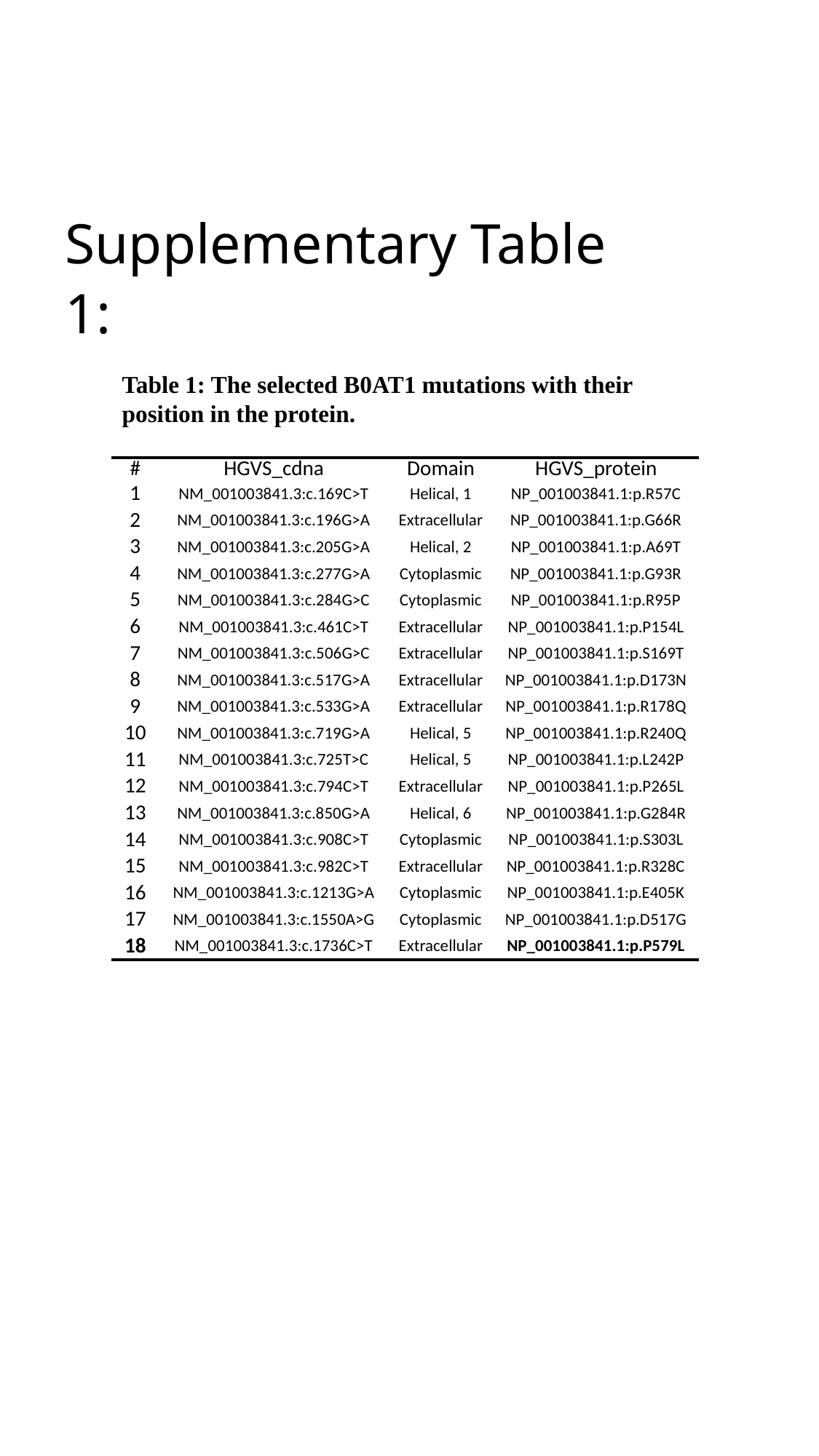

Supplementary Table 1:
Table 1: The selected B0AT1 mutations with their position in the protein.
| # | HGVS\_cdna | Domain | HGVS\_protein |
| --- | --- | --- | --- |
| 1 | NM\_001003841.3:c.169C>T | Helical, 1 | NP\_001003841.1:p.R57C |
| 2 | NM\_001003841.3:c.196G>A | Extracellular | NP\_001003841.1:p.G66R |
| 3 | NM\_001003841.3:c.205G>A | Helical, 2 | NP\_001003841.1:p.A69T |
| 4 | NM\_001003841.3:c.277G>A | Cytoplasmic | NP\_001003841.1:p.G93R |
| 5 | NM\_001003841.3:c.284G>C | Cytoplasmic | NP\_001003841.1:p.R95P |
| 6 | NM\_001003841.3:c.461C>T | Extracellular | NP\_001003841.1:p.P154L |
| 7 | NM\_001003841.3:c.506G>C | Extracellular | NP\_001003841.1:p.S169T |
| 8 | NM\_001003841.3:c.517G>A | Extracellular | NP\_001003841.1:p.D173N |
| 9 | NM\_001003841.3:c.533G>A | Extracellular | NP\_001003841.1:p.R178Q |
| 10 | NM\_001003841.3:c.719G>A | Helical, 5 | NP\_001003841.1:p.R240Q |
| 11 | NM\_001003841.3:c.725T>C | Helical, 5 | NP\_001003841.1:p.L242P |
| 12 | NM\_001003841.3:c.794C>T | Extracellular | NP\_001003841.1:p.P265L |
| 13 | NM\_001003841.3:c.850G>A | Helical, 6 | NP\_001003841.1:p.G284R |
| 14 | NM\_001003841.3:c.908C>T | Cytoplasmic | NP\_001003841.1:p.S303L |
| 15 | NM\_001003841.3:c.982C>T | Extracellular | NP\_001003841.1:p.R328C |
| 16 | NM\_001003841.3:c.1213G>A | Cytoplasmic | NP\_001003841.1:p.E405K |
| 17 | NM\_001003841.3:c.1550A>G | Cytoplasmic | NP\_001003841.1:p.D517G |
| 18 | NM\_001003841.3:c.1736C>T | Extracellular | NP\_001003841.1:p.P579L |

## Slide 3
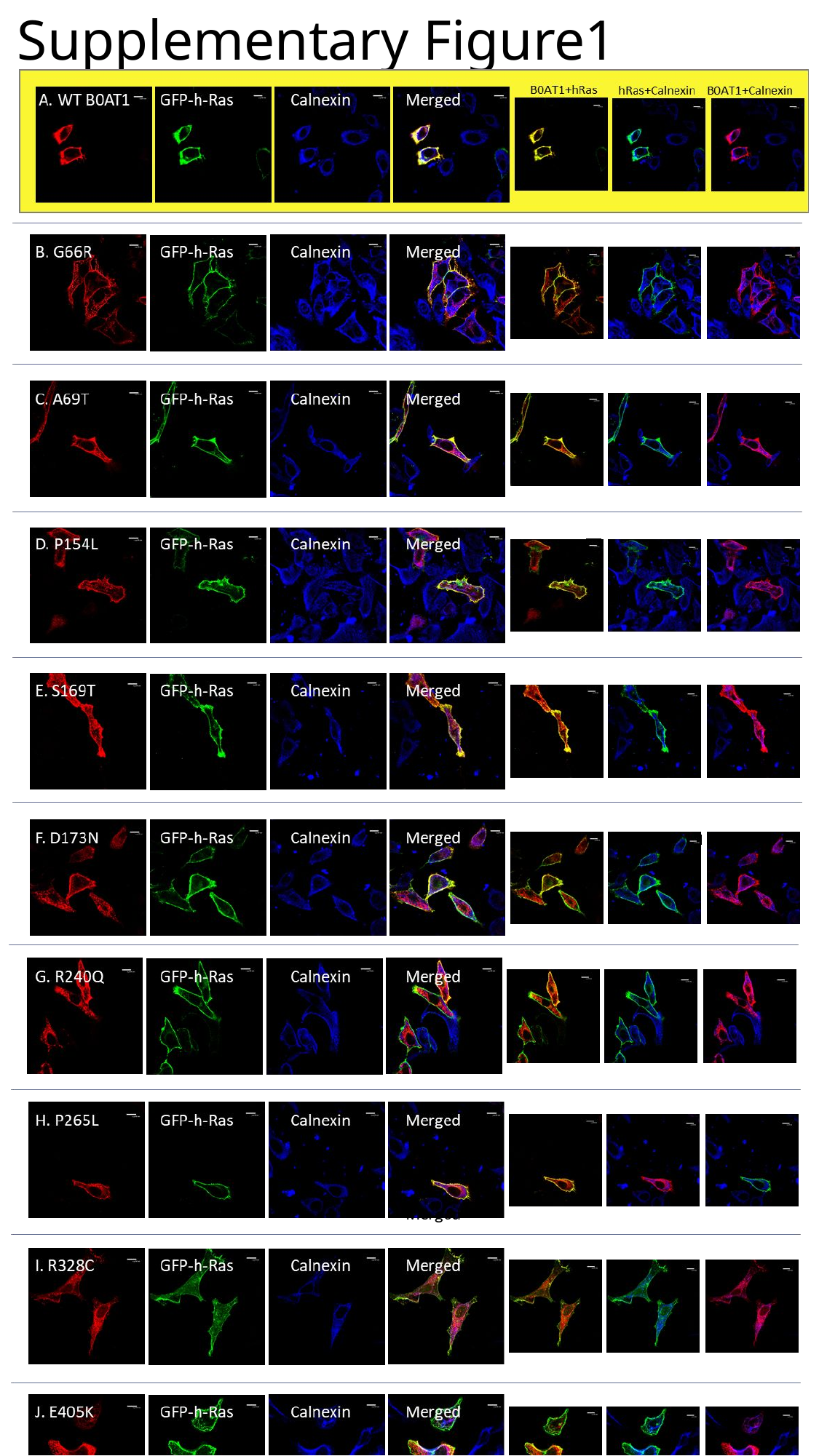

Supplementary Figure1

## Slide 4
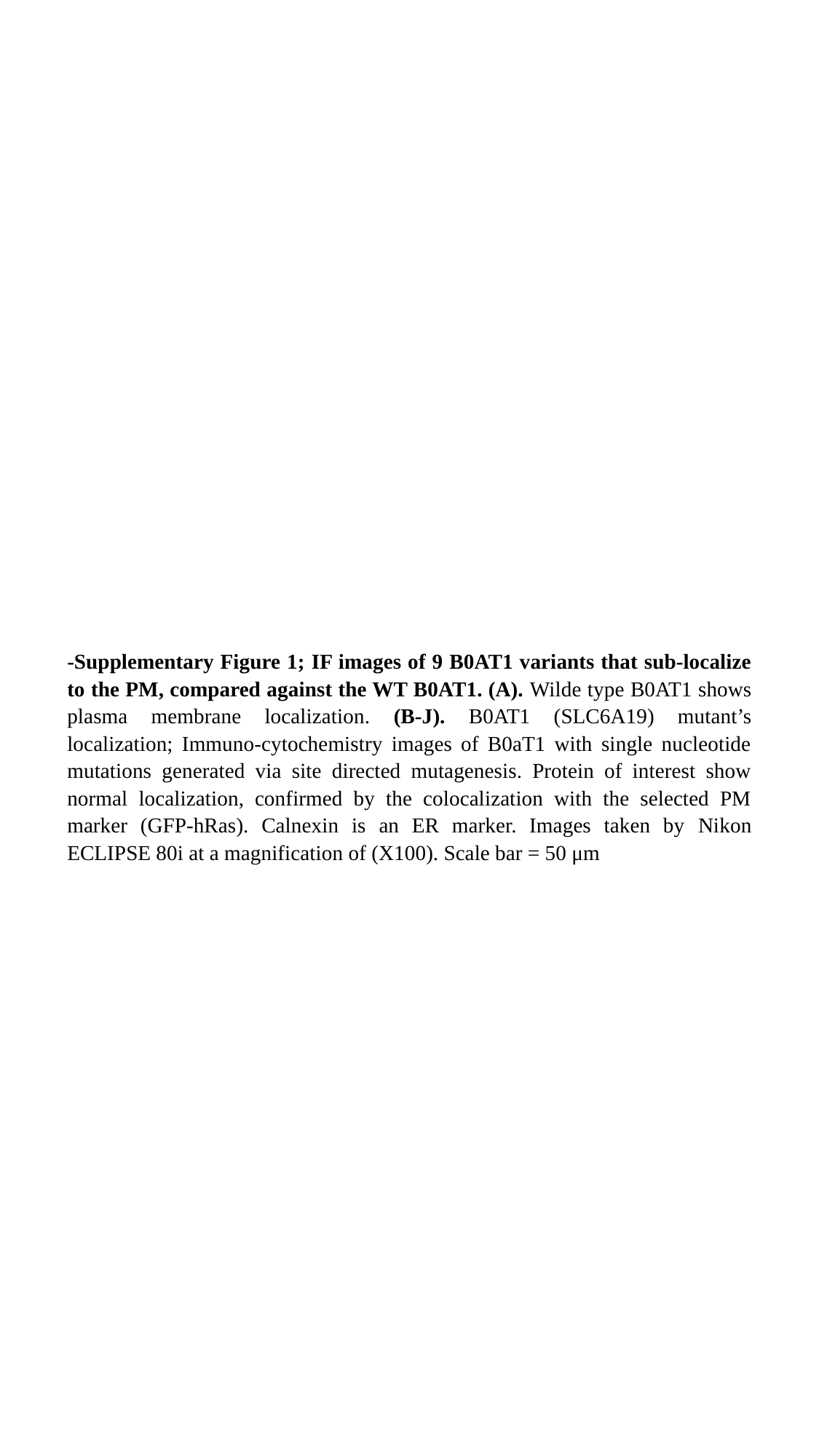

-Supplementary Figure 1; IF images of 9 B0AT1 variants that sub-localize to the PM, compared against the WT B0AT1. (A). Wilde type B0AT1 shows plasma membrane localization. (B-J). B0AT1 (SLC6A19) mutant’s localization; Immuno-cytochemistry images of B0aT1 with single nucleotide mutations generated via site directed mutagenesis. Protein of interest show normal localization, confirmed by the colocalization with the selected PM marker (GFP-hRas). Calnexin is an ER marker. Images taken by Nikon ECLIPSE 80i at a magnification of (X100). Scale bar = 50 μm

## Slide 5
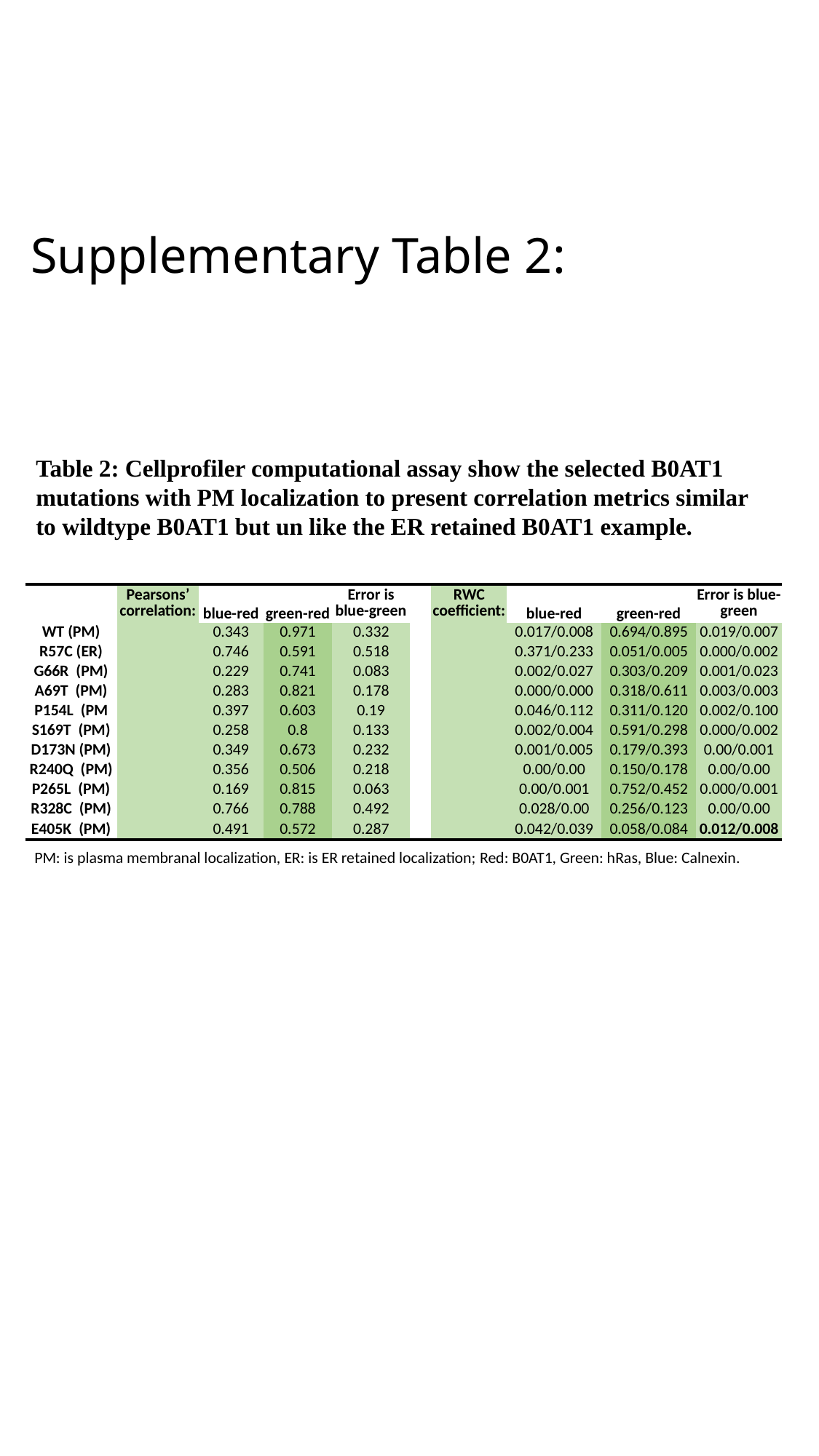

# Supplementary Table 2:
Table 2: Cellprofiler computational assay show the selected B0AT1 mutations with PM localization to present correlation metrics similar to wildtype B0AT1 but un like the ER retained B0AT1 example.
| | Pearsons’ correlation: | blue-red | green-red | Error is blue-green | | RWC coefficient: | blue-red | green-red | Error is blue-green |
| --- | --- | --- | --- | --- | --- | --- | --- | --- | --- |
| WT (PM) | | 0.343 | 0.971 | 0.332 | | | 0.017/0.008 | 0.694/0.895 | 0.019/0.007 |
| R57C (ER) | | 0.746 | 0.591 | 0.518 | | | 0.371/0.233 | 0.051/0.005 | 0.000/0.002 |
| G66R (PM) | | 0.229 | 0.741 | 0.083 | | | 0.002/0.027 | 0.303/0.209 | 0.001/0.023 |
| A69T (PM) | | 0.283 | 0.821 | 0.178 | | | 0.000/0.000 | 0.318/0.611 | 0.003/0.003 |
| P154L (PM | | 0.397 | 0.603 | 0.19 | | | 0.046/0.112 | 0.311/0.120 | 0.002/0.100 |
| S169T (PM) | | 0.258 | 0.8 | 0.133 | | | 0.002/0.004 | 0.591/0.298 | 0.000/0.002 |
| D173N (PM) | | 0.349 | 0.673 | 0.232 | | | 0.001/0.005 | 0.179/0.393 | 0.00/0.001 |
| R240Q (PM) | | 0.356 | 0.506 | 0.218 | | | 0.00/0.00 | 0.150/0.178 | 0.00/0.00 |
| P265L (PM) | | 0.169 | 0.815 | 0.063 | | | 0.00/0.001 | 0.752/0.452 | 0.000/0.001 |
| R328C (PM) | | 0.766 | 0.788 | 0.492 | | | 0.028/0.00 | 0.256/0.123 | 0.00/0.00 |
| E405K (PM) | | 0.491 | 0.572 | 0.287 | | | 0.042/0.039 | 0.058/0.084 | 0.012/0.008 |
PM: is plasma membranal localization, ER: is ER retained localization; Red: B0AT1, Green: hRas, Blue: Calnexin.

## Slide 6
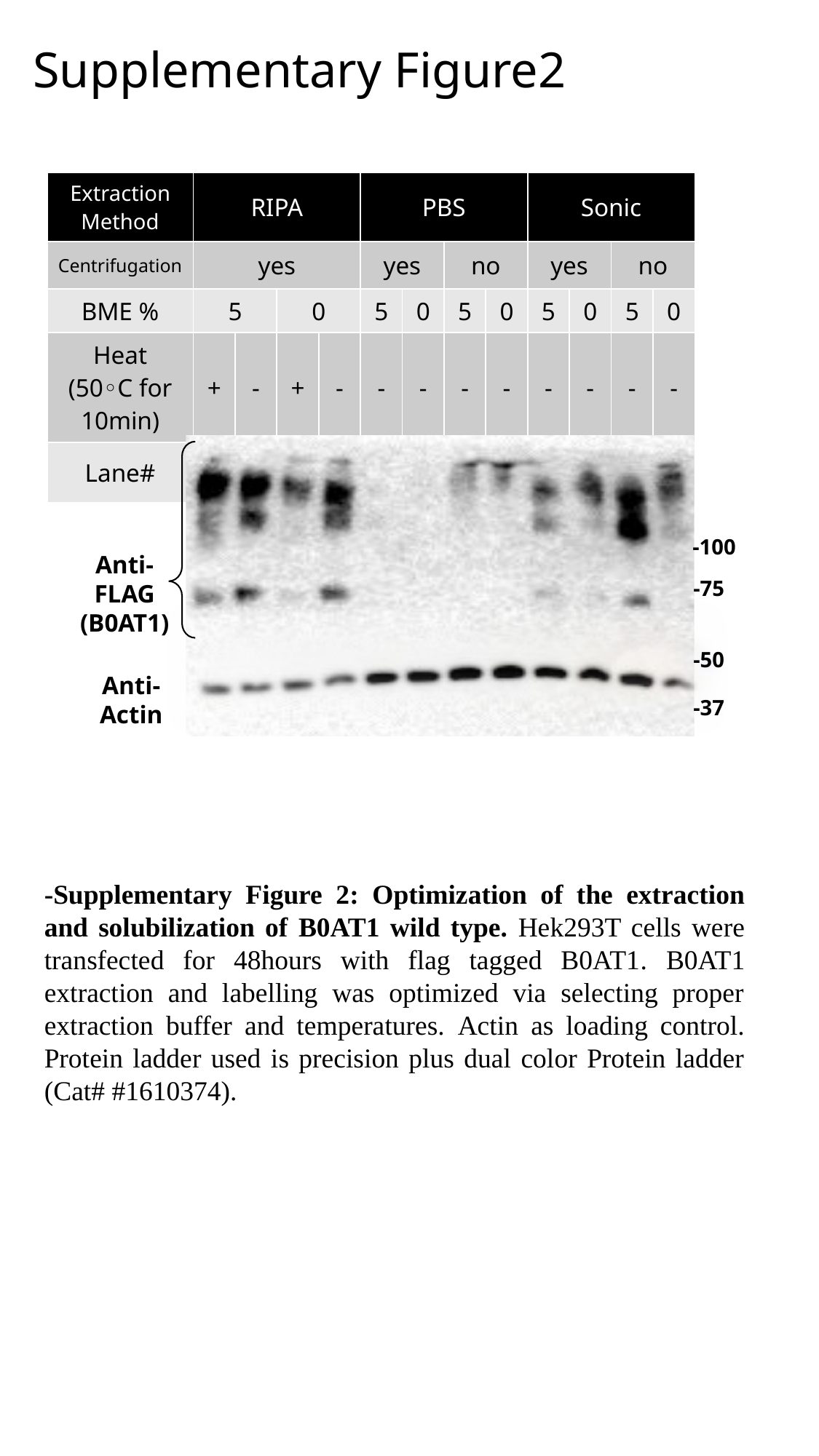

Supplementary Figure2
| Extraction Method | RIPA | | | | PBS | | | | Sonic | | | |
| --- | --- | --- | --- | --- | --- | --- | --- | --- | --- | --- | --- | --- |
| Centrifugation | yes | | | | yes | | no | | yes | | no | |
| BME % | 5 | | 0 | | 5 | 0 | 5 | 0 | 5 | 0 | 5 | 0 |
| Heat (50◦C for 10min) | + | - | + | - | - | - | - | - | - | - | - | - |
| Lane# | 1 | 2 | 3 | 4 | 5 | 6 | 7 | 8 | 9 | 10 | 11 | 12 |
-100
Anti-FLAG
(B0AT1)
-75
-50
Anti-Actin
-37
-Supplementary Figure 2: Optimization of the extraction and solubilization of B0AT1 wild type. Hek293T cells were transfected for 48hours with flag tagged B0AT1. B0AT1 extraction and labelling was optimized via selecting proper extraction buffer and temperatures. Actin as loading control. Protein ladder used is precision plus dual color Protein ladder (Cat# #1610374).

## Slide 7
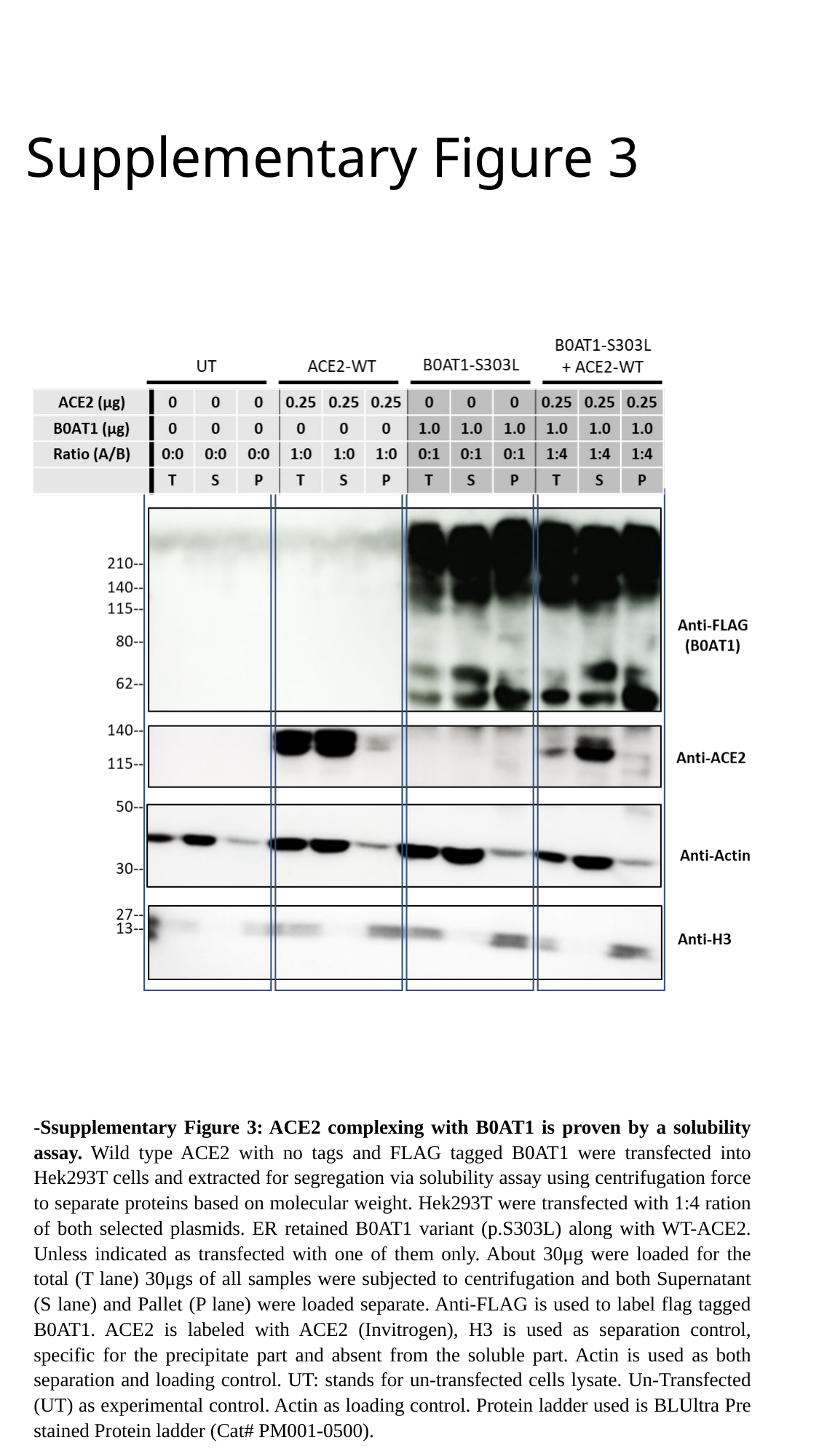

# Supplementary Figure 3
-Ssupplementary Figure 3: ACE2 complexing with B0AT1 is proven by a solubility assay. Wild type ACE2 with no tags and FLAG tagged B0AT1 were transfected into Hek293T cells and extracted for segregation via solubility assay using centrifugation force to separate proteins based on molecular weight. Hek293T were transfected with 1:4 ration of both selected plasmids. ER retained B0AT1 variant (p.S303L) along with WT-ACE2. Unless indicated as transfected with one of them only. About 30μg were loaded for the total (T lane) 30μgs of all samples were subjected to centrifugation and both Supernatant (S lane) and Pallet (P lane) were loaded separate. Anti-FLAG is used to label flag tagged B0AT1. ACE2 is labeled with ACE2 (Invitrogen), H3 is used as separation control, specific for the precipitate part and absent from the soluble part. Actin is used as both separation and loading control. UT: stands for un-transfected cells lysate. Un-Transfected (UT) as experimental control. Actin as loading control. Protein ladder used is BLUltra Pre stained Protein ladder (Cat# PM001-0500).

## Slide 8
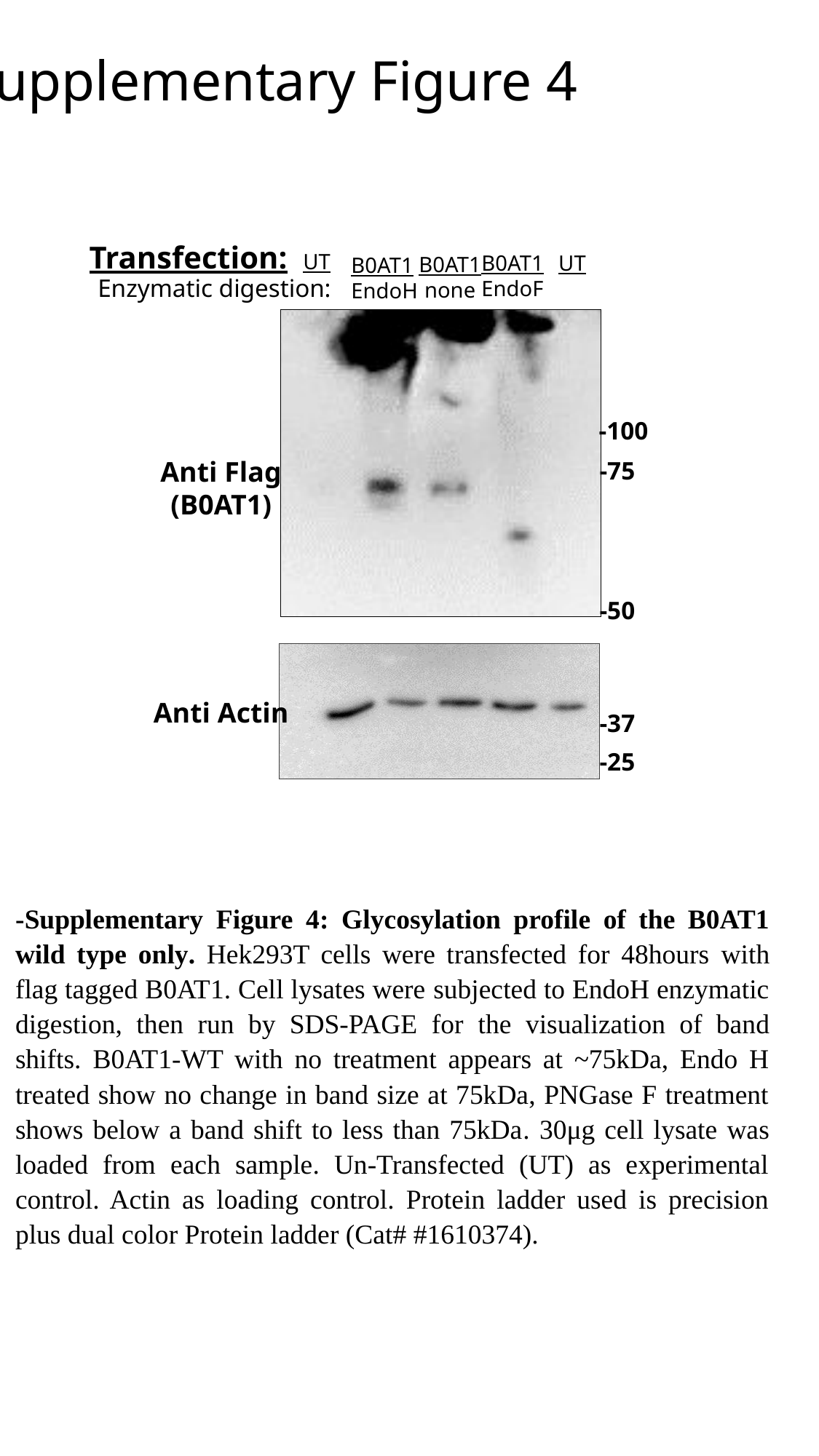

Supplementary Figure 4
Transfection:
UT
UT
B0AT1
EndoF
B0AT1
none
B0AT1
EndoH
Enzymatic digestion:
-100
Anti Flag
(B0AT1)
-75
-50
Anti Actin
-37
-25
-Supplementary Figure 4: Glycosylation profile of the B0AT1 wild type only. Hek293T cells were transfected for 48hours with flag tagged B0AT1. Cell lysates were subjected to EndoH enzymatic digestion, then run by SDS-PAGE for the visualization of band shifts. B0AT1-WT with no treatment appears at ~75kDa, Endo H treated show no change in band size at 75kDa, PNGase F treatment shows below a band shift to less than 75kDa. 30μg cell lysate was loaded from each sample. Un-Transfected (UT) as experimental control. Actin as loading control. Protein ladder used is precision plus dual color Protein ladder (Cat# #1610374).

## Slide 9
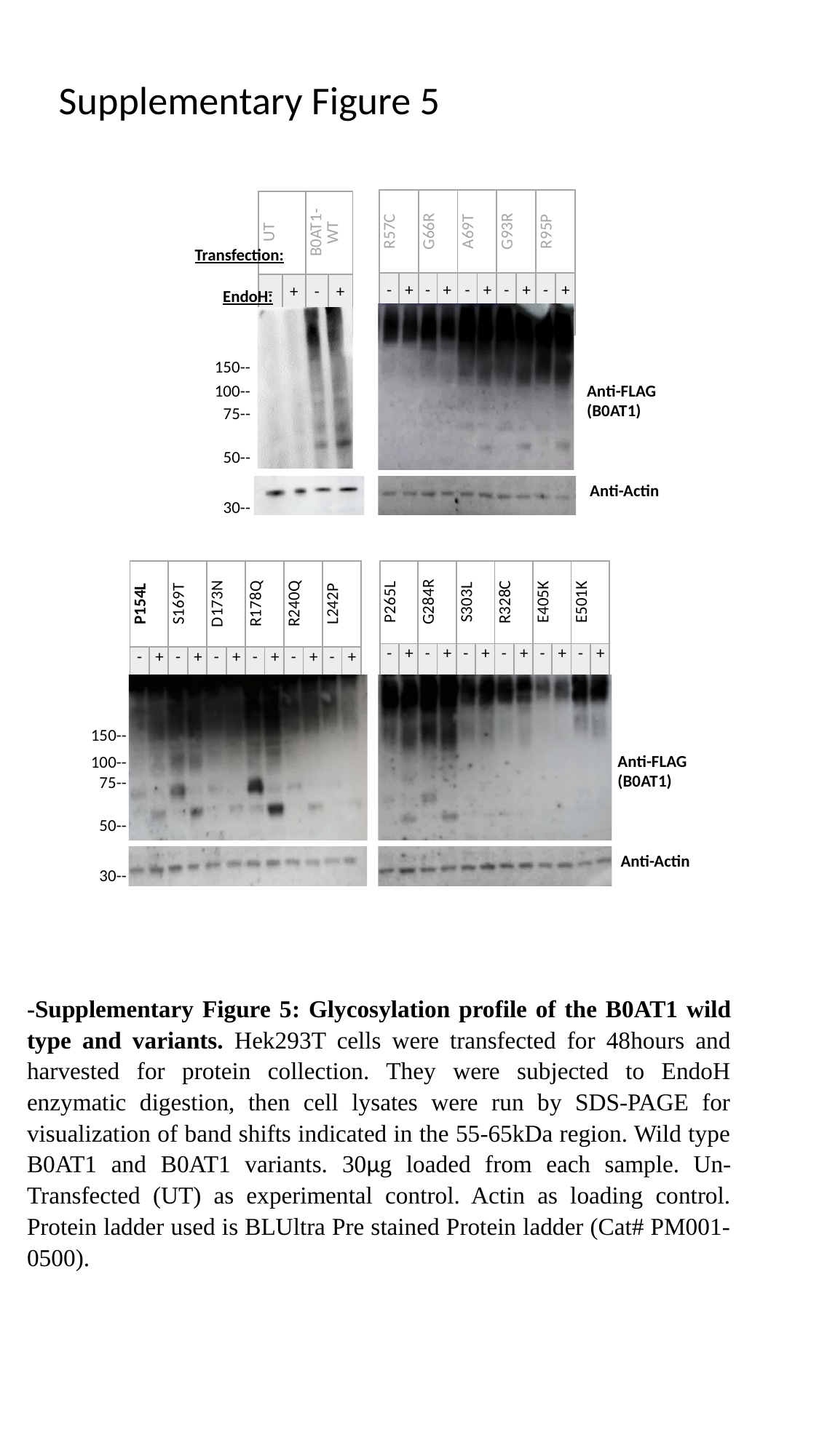

Supplementary Figure 5
| R57C | | G66R | | A69T | | G93R | | R95P | |
| --- | --- | --- | --- | --- | --- | --- | --- | --- | --- |
| - | + | - | + | - | + | - | + | - | + |
| | | | | | | | | | |
| UT | | B0AT1-WT | |
| --- | --- | --- | --- |
| - | + | - | + |
| | | | |
Transfection:
EndoH:
150--
100--
Anti-FLAG
(B0AT1)
75--
50--
Anti-Actin
30--
| P265L | | G284R | | S303L | | R328C | | E405K | | E501K | |
| --- | --- | --- | --- | --- | --- | --- | --- | --- | --- | --- | --- |
| - | + | - | + | - | + | - | + | - | + | - | + |
| | | | | | | | | | | | |
| P154L | | S169T | | D173N | | R178Q | | R240Q | | L242P | |
| --- | --- | --- | --- | --- | --- | --- | --- | --- | --- | --- | --- |
| - | + | - | + | - | + | - | + | - | + | - | + |
| | | | | | | | | | | | |
150--
Anti-FLAG
(B0AT1)
100--
75--
50--
Anti-Actin
30--
-Supplementary Figure 5: Glycosylation profile of the B0AT1 wild type and variants. Hek293T cells were transfected for 48hours and harvested for protein collection. They were subjected to EndoH enzymatic digestion, then cell lysates were run by SDS-PAGE for visualization of band shifts indicated in the 55-65kDa region. Wild type B0AT1 and B0AT1 variants. 30μg loaded from each sample. Un-Transfected (UT) as experimental control. Actin as loading control. Protein ladder used is BLUltra Pre stained Protein ladder (Cat# PM001-0500).
